# Expert consensus on neuro‐musculoskeletal functional reconstruction for post‐stroke spastic hemiplegia

**DOI:** 10.1002/ibra.70032

**Published:** 2026-10-27

**Authors:** Peng Wen, Yihe Wang, Zhongzheng He, Zhizhong Zhu, Chunbing Chen, Kaifeng Wu, Xiaohong Fu, Guoguang Zhao, Liulin Xiong, “Zunyi Brain‐Computer Interface Medical Center” Working Group, Denggui Cheng, Zhaojian Deng, Min Gong, Chong Han, Qijun Jiang, Hang Li, Jianping Li, Shun Li, Yu Li, Minjie Luo, Chiyuan Ma, Yongzhi Shan, Jun Wang, Nana Wang, Qinglan Wang, Ziheng Wang, Zhongying Wei, Guojun Wu, Bo Xiong, Jianguo Xu, Minpeng Xu, Kaihua Yang, Xi Yang, Fan Zhang, Hongtian Zhang, Jing Zhang, Shaofu Zhang, Hongxin Zhao, Min Zhao, Mingjiang Zhu

**Affiliations:** ^1^ Brain‐Computer Interface Medical Center The First People's Hospital of Zunyi (The Third Affiliated Hospital of Zunyi Medical University) Zunyi China; ^2^ Department of Neurosurgery, Xuanwu Hospital Capital Medical University Beijing China; ^3^ Department of Neurosurgery Tianjin Huanhu Hospital Tianjin China; ^4^ Department of Rehabilitation Tianjin Huanhu Hospital Tianjin China; ^5^ Department of Radiology The First People's Hospital of Zunyi (The Third Affiliated Hospital of Zunyi Medical University) Zunyi China; ^6^ Department of Clinical Laboratory The First People's Hospital of Zunyi (The Third Affiliated Hospital of Zunyi Medical University) Zunyi China; ^7^ Department of Neurosurgery The First People's Hospital of Zunyi (The Third Affiliated Hospital of Zunyi Medical University) Zunyi China; ^8^ Cerebrovascular Department The First People's Hospital of Zunyi (The Third Affiliated Hospital of Zunyi Medical University) Zunyi China; ^9^ Cerebrovascular Department Affiliated Hospital of Zunyi Medical University Zunyi China; ^10^ Neurocritical Care Unit The First People's Hospital of Zunyi [The Third Affiliated Hospital of Zunyi Medical University Zunyi China; ^11^ Department of Neurosurgery Affiliated Hospital of North Sichuan Medical College Nanchong China; ^12^ Department of Neurosurgery, Zhujiang Hospital Southern Medical University Guangzhou China; ^13^ Department of Neurosurgery General Hospital of Eastern Theater Command Nanjing China; ^14^ Department of Neurology Yutian County Hospital of Traditional Chinese Medicine Hebei China; ^15^ Department of Neurology The First People's Hospital of Zunyi (The Third Affiliated Hospital of Zunyi Medical University) Zunyi China; ^16^ Department of Neurosurgery, West China Hospital Sichuan University Chengdu China; ^17^ Haihe Laboratory of Brain‐Computer Interaction and Human‐Machine Integration Tianjin University Tianjin China; ^18^ Department of Neurosurgery The Seventh Medical Center of PLA General Hospital Beijing China; ^19^ Department of Pharmacy The First People's Hospital of Zunyi (The Third Affiliated Hospital of Zunyi Medical University) Zunyi China

**Keywords:** brain–computer interface, expert consensus, functional reconstruction, spastic hemiplegia, stroke

## Abstract

Persistent motor dysfunction in post‐stroke spastic hemiplegia may result from impaired voluntary motor control, abnormal muscle overactivity, fixed structural restriction, muscle force imbalance, or their combination. Advances in brain–computer interfaces (BCIs), peripheral nerve surgery, and tendon and soft‐tissue reconstruction have expanded therapeutic options, yet optimal indications and sequencing remain unclear. A multidisciplinary expert panel reviewed current evidence, addressed key clinical questions, and developed consensus recommendations for noninvasive BCI, diagnostic motor nerve block (DNB), selective or hyperselective peripheral nerve surgery, and reconstructive procedures. The consensus emphasizes evaluation of four major components: central voluntary motor impairment, dynamic abnormal muscle overactivity, fixed structural limitations, and muscle force imbalance. Treatment should be guided by the patient's functional goals and the primary factor restricting performance. Task‐oriented motor training remains fundamental when voluntary motor control is impaired, with BCI‐based closed‐loop training as a potential adjunct in selected patients. Focal, functionally significant muscle overactivity caused by abnormal neural drive may be considered for selective or hyperselective peripheral nerve surgery, with DNB assisting patient selection. Fixed structural restrictions may require tendon or soft‐tissue procedures, while muscle force imbalance may benefit from tendon transfer or other dynamic reconstruction. When multiple impairments coexist, combined reconstruction followed by targeted motor retraining and comprehensive reassessment may be appropriate. Overall, neuro‐musculoskeletal functional reconstruction should follow an individualized pathway linking functional goals, dominant pathophysiology, treatment targets, and outcomes. Given the variable evidence quality, no universal treatment sequence or combination strategy is currently supported.

## Graphical abstract

This expert consensus proposes a patient‐centered pathway for functional reconstruction after post‐stroke spastic hemiplegia: “patient goal–pathophysiologic component–treatment target–functional reassessment.” Treatment selection and sequencing across motor retraining, brain–computer interfaces, peripheral nerve surgery, and tendon/soft‐tissue reconstruction should be individualized rather than follow a fixed combination regimen.

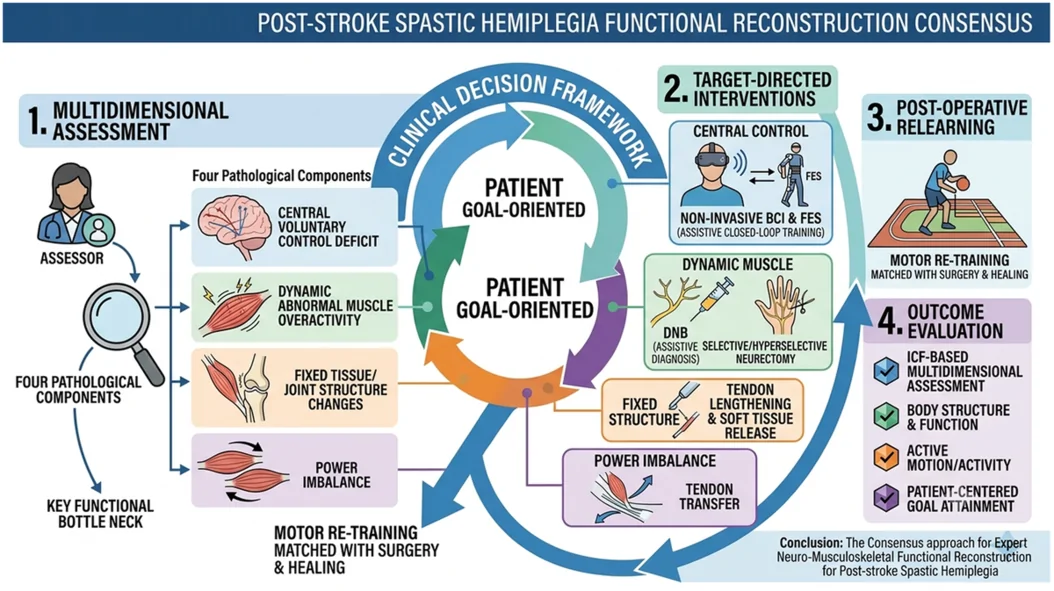

## 1 INTRODUCTION

Stroke remains a leading cause of death and long‐term disability in adults and imposes a particularly heavy burden in China. In 2023, China had an estimated 4.23 million incident strokes, 26.68 million prevalent stroke cases, and 2.05 million stroke‐related deaths.^1^ With continued improvements in acute stroke care and secondary prevention, persistent motor disability and long‐term rehabilitation needs among stroke survivors have become increasingly important determinants of functional independence and long‐term care.

Post‐stroke spasticity and related motor disorders are important contributors to chronic motor disability, although prevalence estimates vary with the definition used, assessment method, time since stroke, and study population. Studies using conventional definitions of post‐stroke spasticity have reported prevalence estimates of approximately 4%–42.6%, with disabling spasticity affecting about 2%–13% of stroke survivors.^2^ A 2026 American Heart Association scientific statement addressing spasticity together with related motor disorders estimated that these conditions may affect approximately 30%–80% of stroke survivors.^3^ Clinical manifestations include abnormal muscle overactivity, impaired selective voluntary motor control, abnormal limb postures, soft‐tissue shortening, and restricted joint motion, with consequent limitations in upper‐limb use, standing and walking, pain management, hygiene, dressing, and daily care. These manifestations cannot be explained by increased muscle tone alone; abnormal neural drive, impaired voluntary motor control, and secondary soft‐tissue changes may contribute simultaneously.^3^

From a neuro‐musculoskeletal reconstruction perspective, persistent post‐stroke motor dysfunction can be analyzed across four interrelated dimensions: impaired central voluntary motor control, dynamic abnormal muscle overactivity, fixed soft‐tissue/joint structural changes, and muscle force imbalance. The same abnormal posture may arise from different pathophysiologic components, and multiple components may coexist in the same patient. In post‐stroke spastic equinovarus foot, for example, abnormal neural drive to the calf muscles, shortening of the triceps surae–Achilles tendon complex, and weakness or imbalance of the ankle dorsiflexors/evertors may all contribute to the deformity.^4^, ^5^ Treatment planning should therefore begin with the patient's functional goal and identification of the predominant impairment limiting that goal, rather than with limb posture or a single measure of muscle tone.

Available interventions target different components of this dysfunction. Noninvasive brain–computer interface (BCI) systems detect brain activity related to motor intention or attempted movement and couple it with functional electrical stimulation (FES), robotic assistance, or other sensory feedback to support closed‐loop motor training.^6^, ^7^, ^8^, ^9^ Diagnostic motor nerve block (DNB) transiently reduces neural drive to a targeted muscle and can help determine the contribution of a dynamic neural component before an irreversible intervention is undertaken.^4^, ^10^ Selective or hyperselective peripheral nerve surgery can provide a more durable reduction in neural drive to selected muscles in appropriately chosen patients with persistent focal muscle overactivity.^11^, ^12^, ^13^, ^14^, ^15^ Tendon lengthening and muscle or fascial release address fixed soft‐tissue restriction, whereas tendon transfer and other dynamic reconstructive procedures may be used to restore muscle force balance.^16^, ^17^, ^18^, ^19^ These interventions act at different levels of the neuro‐musculoskeletal system and are not clinically interchangeable.

These interventions also have different clinical and scientific origins. Vidal's 1973 work on direct brain–computer communication laid an early foundation for modern BCI research.^20^ Peripheral nerve procedures for spasticity date to the early 20th century and became progressively more selective with advances in motor‐branch anatomy and microsurgical technique, including the development of hyperselective neurectomy.^21^, ^22^ DNB has increasingly been used in neuro‐orthopedic assessment before irreversible treatment,^4^, ^10^ whereas tendon lengthening, release, and transfer derive from established principles of reconstructive surgery.^23^ These historical differences do not imply a hierarchy of evidence or treatment priority; rather, they reflect the different pathophysiologic targets of each approach.

Clinical decision‐making should therefore match the patient's functional goals to the predominant pathophysiologic component, define an appropriate treatment target, and determine the sequence of interventions when more than one component requires treatment. Evidence for BCI and surgical functional reconstruction is derived largely from studies conducted within their respective indications, and direct evidence supporting a predetermined combined regimen remains limited. This consensus was developed by a multidisciplinary panel with expertise in rehabilitation medicine, neurology, peripheral nerve and functional neurosurgery, hand surgery and orthopedics, physical therapy, occupational therapy, clinical neurophysiology, and BCI and neuroengineering. It provides recommendations on comprehensive assessment, patient selection, treatment timing and sequencing, postoperative motor relearning, and outcome assessment to support multidisciplinary clinical decision‐making.

## 2 METHODS

### 2.1 Purpose and scope

This document was developed as an evidence‐informed expert consensus. Its reporting was informed by the Reporting Items for Practice Guidelines in Healthcare (RIGHT) statement,^24^ and the consensus process and reporting also drew on the ACcurate COnsensus Reporting Document (ACCORD).^25^ RIGHT was used mainly to guide the reporting of clinical questions, evidence, and recommendations, while ACCORD informed the reporting of expert selection, discussion, voting, and management of disagreement.

This consensus applies primarily to adults with persistent spastic limb motor dysfunction after stroke who require further decision‐making regarding central motor training, spasticity intervention, or neuro‐musculoskeletal functional reconstruction. It focuses on the clinical role, patient selection, boundaries of use, and sequencing of noninvasive BCI, DNB, selective or hyperselective peripheral nerve surgery, and tendon and related soft‐tissue reconstruction.

The document is not intended to replace current standards for secondary stroke prevention, general stroke rehabilitation, or established treatments for spasticity, including oral medication, botulinum toxin injection, and intrathecal therapy. These treatments are discussed only where they influence decisions about neuro‐musculoskeletal reconstruction.

### 2.2 Consensus working group and conflict‐of‐interest management

The consensus working group comprised a steering committee, writing group, evidence appraisal group, and expert panel. The steering committee determined the scope and overall direction of the project. The writing group developed the clinical questions, reviewed and synthesized the evidence, and prepared the manuscript, with support from the evidence appraisal group in literature screening and appraisal of evidentiary support. The expert panel reviewed the proposed statements, participated in multidisciplinary discussion, and voted on the final recommendations and consensus statements.

Experts were purposively selected for their clinical or research experience in areas relevant to post‐stroke spastic motor dysfunction, including rehabilitation, BCI, peripheral nerve and functional neurosurgery, neuro‐orthopedic reconstruction, clinical neurophysiology, neuroengineering, and related fields. Participants were drawn from multiple institutions and regions in China to ensure a range of clinical and technical perspectives. The First People's Hospital of Zunyi initiated and coordinated the project and undertook much of the evidence collation and manuscript preparation; consequently, experts from this institution constituted a larger proportion of the working group. Experts from other centers were included to broaden specialty and institutional representation.

All participants disclosed relevant financial and nonfinancial conflicts of interest before taking part in the consensus process. Experts with a direct conflict concerning a specific item did not participate in its evidence appraisal or final vote. For topics involving BCI, peripheral nerve surgery, or tendon and soft‐tissue reconstruction, discussion also included experts without direct involvement in the development or promotion of the intervention under consideration, to avoid undue influence from a single technical perspective.

Patients and caregivers did not participate directly in the formulation of the clinical questions, evidence appraisal, manuscript drafting, or voting. This is recognized as a limitation of the present consensus, and structured patient and caregiver involvement should be incorporated into future updates. The final expert panel comprised 39 members from 11 institutions (Supplementary Material).

### 2.3 Development of the core clinical questions

The writing group developed the core clinical questions around the main decisions encountered in neuro‐musculoskeletal reconstruction for post‐stroke spastic hemiplegia. The questions were refined through discussion with the steering committee and multidisciplinary experts and were finalized before preparation of the consensus statements. They addressed treatment goals, identification of the predominant pathophysiologic component, choice and timing of intervention, combined or staged reconstruction, postoperative motor retraining, and outcome assessment (Table 1).

**TABLE 1:** Core clinical questions.

No.	Core clinical question
CQ1	How should patient‐centered functional goals be defined before treatment?
CQ2	How should impaired central voluntary motor control and residual potential for active training be identified?
CQ3	How should dynamic abnormal muscle overactivity and the contributing muscles be assessed?
CQ4	How should dynamic neural factors be distinguished from fixed soft‐tissue/joint structural restriction?
CQ5	How should muscle force imbalance and usable donor‐muscle function be assessed?
CQ6	What are the roles and limitations of the Modified Ashworth Scale and Modified Tardieu Scale in decision‐making?
CQ7	What is the role of DNB in decision‐making for selective/hyperselective peripheral nerve surgery?
CQ8	What is the clinical role of noninvasive BCI in post‐stroke motor functional reconstruction?
CQ9	Can BCI‐FES be used as a closed‐loop motor training modality?
CQ10	Can BCI neurophysiologic/decoding metrics be used for surgical screening?
CQ11	Which patients are appropriate candidates for selective/hyperselective peripheral nerve surgery?
CQ12	What are the principal expected benefits and evidence limitations of peripheral nerve surgery?
CQ13	When should tendon lengthening or soft‐tissue release be considered for fixed structural changes?
CQ14	What factors should guide patient selection for tendon transfer?
CQ15	How should individualized single‐stage or staged combined reconstruction be selected in patients with mixed pathophysiologic components?
CQ16	How should motor relearning, outcome assessment, and long‐term follow‐up be conducted after peripheral reconstruction?

Abbreviations: BCI, brain‐computer interface; CQ, clinical question; DNB, diagnostic motor nerve block; FES, functional electrical stimulation.

### 2.4 Literature search and evidence synthesis

Structured literature searches were conducted for the prespecified clinical questions. PubMed was used as the primary database for English‐language literature, and the China National Knowledge Infrastructure (CNKI) for Chinese‐language literature. The final archived PubMed search was conducted on August 30, 2026 and covered seven topic areas: comprehensive assessment, noninvasive BCI rehabilitation, BCI combined with FES, DNB, selective or hyperselective peripheral nerve surgery, tendon or soft‐tissue reconstruction, and postoperative motor retraining. The PubMed search covered publications from January 1, 2016 through August 30, 2026.

The exact date and complete search history of the earlier CNKI search could not be verified from the archived materials and were therefore not reconstructed retrospectively. The archived CNKI records were audited directly, with details provided in the Supplementary Material. Because this work was developed as an evidence‐informed expert consensus rather than a de novo systematic review for each clinical question, separate systematic searches were not undertaken in Embase, the Cochrane Library, Web of Science, Wanfang Data, or SinoMed. Database searches were supplemented by citation tracking from relevant guidelines, consensus documents, systematic reviews, and key primary studies, as well as references suggested by panel members. Earlier landmark publications were retained where necessary to clarify terminology, historical foundations, established reconstructive principles, or long‐term clinical experience. Detailed search strategies, retrieval results, deduplication procedures, and the evidence‐identification audit are provided in the Supplementary Material.

The evidence considered included clinical practice guidelines and consensus documents, systematic reviews and meta‐analyses, randomized controlled trials, comparative studies, and relevant observational studies. In areas where comparative evidence was limited, particularly peripheral nerve surgery and tendon or soft‐tissue reconstruction, case series and long‐term follow‐up studies were also considered, with their methodological limitations taken into account.

Certainty of evidence was not formally graded using the Grading of Recommendations Assessment, Development and Evaluation (GRADE) approach. Instead, the evidence appraisal group considered study design, risk of bias, consistency, directness, precision, and the overall amount of evidence. For recommendations involving a specific clinical action, evidentiary support was described as “substantial,” “limited,” “very limited,” or “no direct evidence.” These categories were used to guide the wording of recommendations and were not intended to correspond to GRADE certainty levels. Where direct evidence was insufficient for a specific action recommendation, but the issue remained clinically important, the panel formulated a consensus statement and described the main evidence and limitations in the accompanying text.

### 2.5 Development of recommendations and consensus statements

The writing group drafted the recommendations and consensus statements from the evidence synthesis and circulated them to panel members for written review before the consensus meeting. During the meeting, each item was introduced together with its supporting evidence, intended scope, and main areas of uncertainty. This was followed by open discussion and independent electronic voting.

Panel members scored each item on a 9‐point scale. Scores of 1–3 indicated disagreement, 4–6 uncertainty or a need for revision, and 7–9 agreement. Consensus was defined a priori as at least 80% of votes in the 7–9 range and fewer than 15% in the 1–3 range. An item with at least 80% agreement but 15% or more disagreement was regarded as showing substantive disagreement and did not meet the consensus criterion. Items that failed to reach consensus, or that were substantially revised after discussion, were modified and submitted for a second vote. Items that still failed to meet the criterion after the second vote were excluded.

For each vote, the number of voters, proportions scoring 7–9 and 1–3, median score, and interquartile range were recorded electronically. The original item‐level voting export was no longer available during final manuscript assembly, so exact item‐level distributions and round‐specific voting data could not be verified and were not reconstructed retrospectively. The Supplementary Material reports the prespecified voting rule and the final disposition of retained items and identifies the unavailable raw voting data as a methodological limitation.

This consensus does not use “strong” or “weak” recommendation terminology or an A/B/C/D grading system. Recommendations are expressed mainly as “recommend,” “may be considered,” or “do not recommend.” Their wording reflects the available evidence, expected benefits and risks, clinical feasibility, and expert judgment. Expert agreement was not regarded as a substitute for evidentiary strength. Where evidence was limited, the text specifies the conditions under which an intervention may be considered and the limits of extrapolation.

## 3 CORE TERMINOLOGY AND BASIC PRINCIPLES

### 3.1 Post‐stroke spastic motor dysfunction

Abnormal limb posture and functional impairment after post‐stroke spastic hemiplegia rarely result from spasticity alone. Classic neurophysiologic definitions describe spasticity primarily as a velocity‐dependent increase in stretch reflexes associated with hyperexcitability of the stretch reflex.^26^ The later concept of “spastic paresis” broadened this view by emphasizing the interaction between impaired voluntary motor control, abnormal muscle overactivity, and secondary changes in muscles and other soft tissues.^27^ In clinical practice, patients may therefore present with various combinations of impaired selective voluntary control, weakness, abnormal synergies, spasticity or other forms of muscle overactivity, muscle‐tendon shortening, joint structural changes, and muscle force imbalance.^3^, ^4^, ^5^ In this consensus, the term “spastic motor dysfunction” is used to describe this broader clinical condition and does not imply that all motor impairment is attributable to spasticity in its narrow neurophysiologic sense.

### 3.2 Four dimensions of clinical assessment

For clinical reasoning and treatment planning, this consensus considers post‐stroke spastic motor dysfunction across four interrelated dimensions. (1) Impaired central voluntary motor control refers to a reduced ability to generate, select, and regulate purposeful voluntary movement. It may include inadequate active recruitment, impaired selective motor control, and abnormal movement synergies. (2) Dynamic abnormal muscle overactivity refers to excessive or inappropriate muscle activity driven by abnormal neural input. This includes spasticity as well as other involuntary muscle activity that becomes apparent with posture, movement, or task performance. (3) Fixed soft‐tissue or joint structural changes refer to persistent restriction of passive motion arising from changes in the muscle‐tendon unit, fascia, joint capsule, or other periarticular structures. Such restriction is not fully corrected by reducing abnormal neural drive alone. (4) Muscle force imbalance refers to a mismatch in the magnitude, direction, or timing of forces acting across a joint, such that available voluntary muscle force cannot be translated effectively into the desired movement. These dimensions often coexist, and their relative contribution may change over time or after treatment. The purpose of the framework is to help identify the predominant impairment or combination of impairments limiting the patient's functional goal and thereby define an appropriate treatment target. It is not intended as an exhaustive classification of post‐stroke disability. Cognition, communication, sensation and perception, pain, endurance, medical stability, and the patient's ability to participate in rehabilitation remain important contextual factors and should be assessed separately.

Expert Consensus Statement 1: Clinical assessment and treatment‐target identification should consider four principal dimensions: impaired central voluntary motor control, dynamic abnormal muscle overactivity, fixed soft‐tissue or joint structural changes, and muscle force imbalance.

Expert Consensus Statement 2: These dimensions may coexist and interact within the same patient and should not be regarded as mutually exclusive diagnostic categories.

Expert Consensus Statement 3: The four‐dimensional framework is intended to support clinical reasoning and treatment‐target identification. It has not been prospectively validated as a predictive model.

### 3.3 Noninvasive BCI

BCI detects and interprets central nervous system activity related to motor intention or attempted movement and converts it into signals that can drive or modulate external feedback. In this consensus, the focus is on noninvasive BCI for motor rehabilitation, particularly systems using electroencephalographic signals in closed‐loop training with FES, robotic assistance, visual feedback, or other forms of sensory feedback.^6^ BCI is considered a rehabilitation approach rather than a stand‐alone tool for determining surgical candidacy. Classification accuracy or any single neurophysiologic measure should therefore not be used in isolation to select patients for surgery.

### 3.4 Selective and hyperselective peripheral nerve surgery

In this consensus, selective and hyperselective peripheral nerve surgery refers to procedures that partially interrupt or selectively denervate motor nerves or motor branches supplying target muscles, with the aim of achieving a sustained reduction in abnormal neural drive. Terminology varies across the literature, and terms such as selective neurotomy, selective neurectomy, and hyperselective neurectomy are not always used consistently. Hyperselective neurectomy (HSN) represents one technique within this broader group. When a specific procedure is reported or considered, the target nerve, level of intervention, motor branches treated, and operative technique should be specified rather than relying on the procedure name alone.^12^, ^13^, ^14^, ^15^

Selective dorsal rhizotomy (SDR) is not included within this definition. Unlike selective peripheral motor denervation, SDR reduces spasticity by interrupting selected sensory afferent fibers at the level of the dorsal spinal roots. Its anatomic target and mechanism are therefore distinct. Most evidence for SDR concerns cerebral palsy; studies in adults are less common, and direct evidence in post‐stroke spasticity remains particularly limited.^28^ Given the population and reconstructive focus of this consensus, SDR is outside the scope of the present recommendations.

Percutaneous cryoneurolysis has also been explored as a peripheral nerve intervention for focal spasticity. A 2026 systematic scoping review identified 25 studies but no published randomized controlled trials, and the certainty of evidence for efficacy outcomes was considered very low.^29^, ^30^ Because patient selection, target‐nerve selection, procedural parameters, long‐term benefit and safety, and the role of cryoneurolysis within current spasticity pathways remain insufficiently defined, this consensus does not issue a specific recommendation for it and does not regard it as an equivalent substitute for DNB or selective/hyperselective peripheral nerve surgery.

### 3.5 Tendon and soft‐tissue functional reconstruction

Tendon and soft‐tissue functional reconstruction in this consensus includes tendon lengthening, muscle/fascial release, tendon transfer, and other soft‐tissue procedures used to correct fixed structural restriction or improve muscle force balance. Tendon lengthening and soft‐tissue release primarily address fixed structural restriction, whereas tendon transfer changes the insertion and line of pull of available muscle force so that existing power generates a new functional moment. Because these procedures have different therapeutic objectives, they should not be grouped generically as treatments to “reduce spasticity.”^16^, ^17^, ^18^, ^19^

## 4 COMPREHENSIVE ASSESSMENT BEFORE FUNCTIONAL RECONSTRUCTION

Before treatment targets are identified using the four‐dimensional framework, clinicians should first confirm medical stability and assess factors that may affect treatment feasibility or rehabilitation participation, including cognition and communication, attention, sensation and perception, pain, and endurance. Comprehensive assessment should then be guided by the patient's functional goals and combine clinical examination with reproducible task‐based and functional measures. When necessary, DNB, dynamic electromyography (EMG), gait analysis, and imaging may provide additional information.

Musculoskeletal ultrasound and shear wave elastography (SWE) can provide quantitative information on selected aspects of muscle morphology and passive mechanical properties. Systematic reviews have shown altered mechanical properties of affected muscles after stroke, and a recent meta‐analysis reported a moderate overall correlation between SWE measurements and clinical spasticity scales.^31^, ^32^ These techniques may therefore complement clinical assessment, particularly when passive muscle stiffness or structural changes are of interest, but should not be used as substitutes for established clinical evaluation. Examination protocols remain insufficiently standardized, and there is currently no evidence that ultrasound or SWE alone can reliably distinguish pathophysiologic components, determine treatment selection, or improve patient‐centered outcomes. Routine use is therefore not required, and no standalone decision threshold is recommended. Ultrasound or SWE may be used selectively when it addresses a specific structural or mechanical question and appropriate expertise and resources are available.

### 4.1 Define patient‐centered functional goals

Assessment for neuro‐musculoskeletal functional reconstruction should begin with the patient's most important and meaningful functional goals, rather than with the selection of a particular intervention. Relevant goals may include active upper‐limb use, grasp and release, standing and walking, foot placement, pain relief, hygiene, dressing, footwear or orthosis fit, appearance, and reduced caregiver burden. GAS may be used to define and track individualized goals, provided that these goals are specific, clinically meaningful, and established before treatment.^33^, ^34^

### 4.2 Assess residual voluntary motor capacity

An important consideration in active functional reconstruction is whether sufficient voluntary motor capacity remains to support useful movement once peripheral constraints are reduced. Residual voluntary motor function should be assessed by clinical observation, active range of motion (ROM), selective motor control, muscle strength, and task performance. Surface or dynamic EMG and other neurophysiologic assessments may provide additional information when needed. The Fugl‐Meyer Assessment (FMA) can be used to characterize overall motor impairment, but no single FMA threshold should be used to determine candidacy for peripheral nerve or tendon surgery.

### 4.3 Assess abnormal muscle overactivity

Assessment of abnormal muscle overactivity should determine whether it contributes meaningfully to the abnormal posture or limitation of the patient's target function and, where possible, identify the muscles primarily responsible. The Modified Ashworth Scale (MAS) may be used to document resistance to passive movement and changes over time. However, the measured resistance reflects both neural and non‐neural factors, and MAS alone cannot distinguish abnormal neural drive from muscle‐tendon shortening or joint stiffness.^35^, ^36^ The Modified Tardieu Scale (MTS), which evaluates muscle responses at different stretch velocities, may provide additional information about the dynamic component of muscle overactivity. Its measurement properties also have limitations, however, and it should not be regarded as a definitive method for distinguishing spasticity from fixed structural restriction.^37^, ^38^

Expert Consensus Statement 4: The MAS may be used to document resistance to passive movement and its change over time, but should not be used alone to determine the underlying mechanism of motor dysfunction. In particular, it does not distinguish abnormal neural drive from non‐neural restriction or assess residual voluntary motor control and muscle force balance. Decisions regarding peripheral nerve surgery, tendon lengthening, or tendon transfer should therefore not be based on a single MAS score.

### 4.4 Identify fixed structural restriction

Fixed structural restriction should be assessed primarily by slow passive joint ROM, muscle‐tendon length, joint status, and end‐feel. Increased resistance during rapid stretch with relatively preserved passive ROM at slow speed suggests that a dynamic neural component is prominent. By contrast, persistent limitation of passive motion during slow examination, or after reduction of dynamic muscle overactivity, suggests an important fixed structural component. A diagnosis of fixed contracture does not require prior treatment with botulinum toxin or permanent peripheral nerve surgery. When dynamic and fixed components cannot be distinguished reliably on clinical examination, and the distinction would alter treatment planning, a reversible intervention such as DNB may be used as an adjunct to clarify their relative contributions.^4^, ^11^

### 4.5 Assess muscle force balance

Muscle strength may be described using the Medical Research Council (MRC) scale, but assessment of muscle force balance should extend beyond a static strength grade. This is particularly important when tendon transfer is being considered. Evaluation should determine whether the proposed donor muscle can be recruited voluntarily and repeatedly, whether its strength is sufficient for the intended function, and whether the target joint has adequate passive ROM. Antagonist overactivity or other competing forces should also be considered, together with the patient's ability to participate in postoperative motor relearning.^18^, ^19^

### 4.6 Identify the principal treatment target

After completing the above assessments, the clinician should identify the factor or combination of factors that most directly limits the patient's predefined functional goal. When one pathophysiologic component clearly predominates, treatment may initially focus on that component. When several components contribute, their relative importance and interaction should be considered in deciding whether interventions should be staged or combined. Because the dominant limiting factor may change after treatment, reassessment should continue throughout the course of functional reconstruction.

Recommendation 1: For patients with persistent post‐stroke spastic limb motor dysfunction that interferes with active limb function, walking, limb posture, hygiene, dressing, pain management, or caregiving, we recommend a multidimensional assessment centered on the patient's specific functional goals. The relative contributions of impaired central voluntary motor control, dynamic abnormal muscle overactivity, fixed structural restriction, and muscle force imbalance should be assessed to identify the principal treatment target.

(Evidence support: substantial)

The clinical features, main assessment approaches, treatment directions, and limitations of interpretation for the four pathophysiologic dimensions are summarized in Table 2.

**TABLE 2:** Clinical identification, assessment, and treatment boundaries for the four major pathophysiologic dimensions.

Pathophysiologic dimension	Clinical features suggesting that it is a major functional bottleneck	Primary assessment	Primary treatment direction	Limits of clinical interpretation
Impaired central voluntary motor control	Marked difficulty performing the target movement actively, with inadequate recruitment, impaired selective motor control, or abnormal synergies; slow passive ROM is relatively preserved, or active control remains markedly limited after peripheral restrictions are reduced.	Active joint motion, selective motor control, strength, FMA, and performance of target tasks; surface/dynamic EMG or other neurophysiologic assessment when needed.	Task‐oriented and task‐specific training as the foundation; BCI, FES, robotics, or other feedback technologies may be added in appropriate patients.	Central motor training does not replace treatment of a clear fixed contracture or major mechanical restriction; BCI or a single neurophysiologic metric should not be used as a surgical indication or exclusion criterion.
Dynamic abnormal muscle overactivity	Abnormal posture or passive resistance varies with stretch velocity, posture, or task; greater ROM is available with slow stretch; observable changes in posture, joint motion, or target tasks occur after DNB.	Dynamic clinical examination; MAS/Modified Tardieu; DNB, dynamic EMG, or gait analysis when needed.	Select reversible treatments such as rehabilitation, medication, or botulinum toxin according to patient goals; persistent, focal limitations despite appropriate nonsurgical treatment may prompt evaluation for selective/hyperselective peripheral nerve surgery.	If substantial passive restriction remains after abnormal neural drive is reduced, fixed structural change should be assessed further; reduced muscle tone does not equal recovery of active function.
Fixed soft‐tissue/joint structural changes	Stable ROM restriction, muscle‐tendon shortening, or abnormal end‐feel persists during sufficiently slow passive movement or after dynamic abnormal muscle activity is reduced; substantial mechanical restriction remains after DNB.	Slow passive ROM, muscle‐tendon length, joint status, and end‐feel; musculoskeletal ultrasound or other imaging and/or DNB may be added when useful.	Consider tendon lengthening, muscle/fascial release, or other structural reconstruction according to the specific abnormality.	Structural reconstruction primarily improves mechanical conditions and does not replace central motor‐control training; fixed contracture should not be inferred from MAS alone.
Muscle force imbalance	Useful voluntary strength is present, but the direction of muscle action, activation timing, or agonist‐antagonist balance is unfavorable during the target movement; the donor muscle can be recruited repeatedly, but existing power is not effectively translated into the target action.	Donor‐muscle recruitment and stability, strength, dynamic function, ROM, and antagonist status; dynamic EMG or gait analysis when needed.	Tendon transfer or other dynamic reconstruction may be considered when appropriate, combined with postoperative motor relearning.	A single MRC grade or FMA threshold should not determine candidacy; fixed structural restriction and abnormal neural drive must still be identified and addressed separately.

Abbreviations: BCI, Brain‐computer interface; EMG, electromyography; FMA, Fugl‐Meyer Assessment; FES, functional electrical stimulation; MRC, Medical Research Council; MAS, Modified Ashworth Scale; ROM, range of motion.

### 4.7 General principles for the timing of intervention and clinical situations requiring caution

The timing of functional reconstruction after stroke should not be determined by a fixed post‐stroke interval alone. More important considerations include the treatment objective, the pathophysiologic component being targeted, and whether the proposed intervention is reversible. Noninvasive or reversible approaches such as BCI and DNB may be considered once the relevant clinical prerequisites are met. BCI has been studied across early, subacute, and chronic stages after stroke,^7^, ^8^, ^9^ while no minimum post‐stroke interval has been established for DNB when it is needed to address a specific clinical decision.^4^, ^10^

Irreversible or structural procedures are usually considered after an appropriate period of rehabilitation and, where indicated, reversible treatment, when the pattern of motor impairment or structural restriction is sufficiently stable to define realistic treatment goals and a postoperative rehabilitation plan. In adults with acquired brain injury, approximately 6 months is commonly used as a practical reference point, and some reports refer to intervals of 9–12 months; these time points should not be interpreted as validated thresholds for surgery.^39^, ^40^ Earlier surgical evaluation may be appropriate when pain, hygiene problems, caregiver burden, or progressive fixed contracture have become clinically important. In such circumstances, the expected benefits and risks should be considered on an individual basis within a multidisciplinary assessment.

No uniform, validated set of absolute contraindications applies across all interventions considered in this consensus. Suitability also depends on general medical and anesthetic risk, local procedural factors, and the patient's ability to participate in postoperative rehabilitation. The recommendations, therefore, distinguish between principal clinical scenarios in which an intervention may be appropriate and situations in which it should be avoided or used with particular caution. Individual procedures should also follow the relevant standards for anesthesia, surgery or intervention, and device safety.

The principal appropriate scenarios, timing considerations, and situations requiring avoidance or caution for the major interventions are summarized in Table 3.

**TABLE 3:** Principal indications, timing, and situations requiring avoidance or caution for major techniques.

Technique	Principal appropriate scenario	Timing	Situations in which use is inappropriate or warrants caution
Noninvasive BCI	Post‐stroke motor impairment with a goal of further active training and the ability to understand and participate; used as an adjunct to task‐oriented rehabilitation.	May be assessed once the patient is medically stable and can participate actively; no fixed number of months is required. Multicenter randomized trials have included patients within 1 month of stroke, and studies also include subacute and chronic phases.	Severe impairment of consciousness, cognition, attention, or communication that prevents task completion; inability to tolerate training or equipment; skin problems at signal‐acquisition sites that interfere with electrode use. If combined with FES, electrical‐stimulation safety must be assessed separately.
BCI‐FES	BCI training in which a closed loop is created through peripheral sensorimotor feedback, with target muscles/peripheral structures suitable for FES.	Same general timing as BCI, with no uniform start time; FES tolerance and target‐muscle condition should also be considered.	In addition to BCI‐related limitations, FES should not be used when electrical stimulation is unsafe or impractical; assess according to the device and individual patient factors.
DNB	Need to distinguish dynamic abnormal neural drive from fixed structural restriction, identify contributing muscles, or simulate potential benefit and functional trade‐offs before selective/hyperselective peripheral nerve surgery.	May be performed whenever a clear clinical decision question exists and the result could change treatment; no fixed interval after stroke is required.	Infection at the intended block site, known allergy to the local anesthetic, or unacceptable puncture/bleeding risk; decision value is limited if comparable clinical or functional assessment cannot be completed before and after the block.
Selective/hyperselective peripheral nerve surgery	Persistent, focal, goal‐relevant dynamic abnormal muscle overactivity that continues to substantially affect function, symptoms, posture, or care despite standard rehabilitation and appropriate nonsurgical treatment, with an expected net benefit from durable reduction of target‐muscle neural drive.	Usually considered after recovery trajectory and spasticity pattern are relatively stable and reversible treatment has been adequately assessed; 6 months may be used as an empirical reference, not a hard threshold.	Principal limitation is fixed contracture rather than dynamic neural drive; reducing target‐muscle tone may substantially impair a useful compensatory function; the relevant muscle/target nerve is unclear; motor disorder pattern is clearly unstable; patient cannot undergo surgery or postoperative rehabilitation.
Tendon lengthening/muscle‐fascial release	Clear fixed shortening of muscle‐tendon or soft tissue causing a mechanical limitation in passive ROM, hygiene, pain control, footwear/orthosis fit, or subsequent active training.	Consider when the fixed structural restriction is stable and has become a major bottleneck; surgical evaluation generally occurs after the recovery trajectory and structural state are relatively stable, without a uniform month‐based threshold.	Abnormality is mainly reversible dynamic spasticity without clear fixed shortening; excessive lengthening may sacrifice useful strength; local soft‐tissue or general medical condition is unsuitable for surgery, postoperative immobilization, or rehabilitation.
Tendon transfer	A donor muscle can be recruited repeatedly and has useful strength, but its direction of action/moment is unfavorable; the target joint has adequate passive ROM and the patient can participate in postoperative motor relearning.	Usually considered after neurologic recovery and the force pattern are relatively stable; in adults with acquired brain injury this often occurs in the chronic phase, but no validated uniform time threshold exists.	Unstable donor‐muscle recruitment or inadequate strength; untreated fixed contracture of the target joint; uncontrolled abnormal antagonist drive; continually changing motor pattern; inability to complete postoperative immobilization, training, or new motor‐mapping learning.

Abbreviations: BCI, brain‐computer interface; DNB, diagnostic motor nerve block; FES, functional electrical stimulation; ROM, range of motion.

## 5 ROLE OF NONINVASIVE BCI IN FUNCTIONAL RECONSTRUCTION

### 5.1 BCI as an adjunct to standard rehabilitation

Noninvasive BCI detects brain activity related to motor intention or attempted movement and links it to visual, FES, robotic, or other forms of feedback, creating a closed sensorimotor loop during active training. Current evidence has evaluated BCI mainly as an adjunct to conventional post‐stroke motor rehabilitation.

A 2026 Cochrane review included 43 studies involving 1628 adults with stroke. Compared with usual care, BCI may provide a small improvement in upper‐limb motor function, while evidence for lower‐limb function and activities of daily living remains limited or uncertain. Compared with sham BCI, no consistent benefit in upper‐limb motor function was demonstrated, and the overall certainty of evidence was low to very low.^7^ A 2025 meta‐analysis of 21 randomized controlled trials (RCTs) involving 886 participants reported a mean between‐group improvement of 3.69 points on the Fugl‐Meyer Assessment–Upper Extremity (FMA‐UE) with BCI training (95% confidence interval [CI], 2.41–4.96), although heterogeneity was substantial (*I* ^2^ = 70%).^8^

The difference in evidence ratings between these reviews likely reflects differences in included studies, comparators, outcomes, and appraisal methods. More importantly, most trials enrolled general post‐stroke rehabilitation populations rather than patients specifically selected for spastic neuro‐musculoskeletal reconstruction. The available evidence is therefore indirect for the reconstructive population addressed in this consensus, and findings from upper‐limb studies should not be assumed to apply to lower‐limb reconstruction.

Recommendation 2: In patients with post‐stroke upper‐limb motor impairment who have a goal of further active motor training and are able to participate, noninvasive closed‐loop BCI training may be considered as an adjunct to standard task‐oriented rehabilitation. (Evidence support: limited.) BCI should not replace standard motor rehabilitation, and treatment should be delivered in medically stable patients by appropriately trained teams with suitable equipment and quality‐control procedures. A fixed post‐stroke interval is not required for initiation.

### 5.2 BCI‐FES and other closed‐loop feedback modalities

BCI may be paired with FES, robotic or exoskeleton‐assisted movement, visual feedback, or other sensory feedback to provide closed‐loop motor training. A 2024 systematic review and meta‐analysis by Ren et al. included 10 randomized controlled trials involving 290 patients with stroke and found that BCI‐FES improved upper‐limb motor function compared with control treatment, with a pooled standardized mean difference (SMD) of 0.50 (95% CI, 0.26–0.73). Evidence regarding the durability of benefit remains limited.^9^

Recommendation 3: BCI‐FES may be considered as an option for closed‐loop upper‐limb motor training after stroke in patients who are suitable for BCI training and peripheral electrical stimulation. (Evidence support: limited.) Treatment should be selected according to the patient's functional goals and practical feasibility and delivered in centers with appropriate equipment, trained personnel, and quality‐control procedures. Because BCI paradigms, decoding methods, FES parameters, training frequency, and treatment duration vary considerably across studies, no uniform technical threshold or treatment regimen is recommended at present.

### 5.3 Limits of clinical interpretation of BCI metrics

BCI classification accuracy, event‐related desynchronization/synchronization (ERD/ERS), movement‐related cortical potentials (MRCP), and related measures can provide information about task‐related brain activity and BCI performance. However, these measures are influenced by attention, cognition, fatigue, task comprehension, signal quality, and algorithm performance. The presence of task‐related brain activity does not indicate that the patient necessarily has the voluntary strength, joint ROM, tendon length, or biomechanical conditions required for the target movement. Conversely, poor decoding performance in a single BCI session should not by itself be taken to exclude the potential for functional reconstruction.

Expert Consensus Statement 5: BCI classification accuracy, ERD/ERS, MRCP, and related measures may provide supplementary information about task‐related brain activity and BCI performance, but should not be used alone as an indication, exclusion criterion, or surgical threshold for peripheral nerve surgery or tendon and soft‐tissue reconstruction.

### 5.4 Patient selection, timing, and safety boundaries

Patient selection for noninvasive BCI should focus on whether the patient can participate meaningfully in active closed‐loop training rather than on a fixed interval after stroke. A 17‐center randomized controlled trial in China enrolled patients with ischemic stroke within 1 month of onset and demonstrated the feasibility of BCI‐assisted training early after stroke.^41^ Systematic reviews and randomized trials have also included patients in the subacute and chronic stages.^7^, ^8^, ^9^ BCI may therefore be considered once the patient is medically and neurologically stable, can understand the training task and maintain sufficient attention, and is able to engage in motor‐intention or attempted‐movement tasks. In patients with severe impairment of consciousness or cognition who cannot understand the task or participate consistently, meaningful closed‐loop training may not be achievable. When BCI‐FES is used, the safety of peripheral electrical stimulation, the condition of the target muscles and skin, and patient tolerance should also be assessed.

## 6 DIAGNOSTIC MOTOR NERVE BLOCK

DNB uses a local anesthetic to temporarily reduce neural drive to a target muscle by blocking its motor nerve or motor branch. Changes in limb posture, passive ROM, active movement, and relevant functional tasks can then be assessed while activity in the target muscle is reduced. The main value of DNB is not to predict surgical success directly, but to provide a reversible means of clarifying the components contributing to the patient's motor dysfunction. It can help determine the contribution of dynamic abnormal muscle overactivity, identify the muscles involved, and reveal fixed contracture, antagonist weakness, or other biomechanical limitations that may become more apparent after the target muscle is temporarily weakened.^4^, ^11^

A 2026 systematic review by Archibold et al. included nine studies, five involving neurotomy and four involving botulinum toxin, all addressing the lower limb. Short‐term changes in spasticity and selected gait measures after DNB showed some concordance with subsequent neurotomy outcomes, whereas prediction of longer‐term outcomes such as strength and joint ROM was less consistent. Block techniques and assessment protocols also varied considerably across studies.^11^ DNB may therefore be particularly useful when the contribution of a dynamic neural component remains uncertain after clinical examination.

DNB can also complement dynamic gait assessment in complex spastic equinus. In a 2022 retrospective study of 40 adults with central nervous system lesions, including 22 patients with stroke, Cormier et al. compared clinical examination, tibial motor nerve block, and instrumented three‐dimensional gait analysis. The three approaches provided overlapping but not identical information, and DNB helped clarify changes in ankle dorsiflexion and the contribution of fixed contracture after temporary reduction of calf‐muscle overactivity.^42^ Because the study included mixed neurologic etiologies, its findings should be applied to patients with stroke with appropriate clinical judgment.

Overall, direct evidence supporting DNB in surgical decision‐making is strongest for lower‐limb reconstruction. Its use in upper‐limb reconstruction is supported by more limited and indirect evidence and should therefore be interpreted in the context of the individual clinical findings and multidisciplinary assessment.

### 6.1 Changes to assess before and after DNB

DNB should be performed and interpreted in relation to a predefined clinical question and the patient's functional goals. Its result should not be reduced to a simple “positive” or “negative” response based only on a change in MAS or a single joint angle. Before the block, clinicians should document the target posture, passive ROM at slow and faster stretch, the muscles thought to contribute to the abnormal posture, available active movement, and relevant goal‐directed tasks. These assessments should be repeated after the block under comparable posture and task conditions whenever possible.

The DNB record should document the clinical question being addressed, the nerve or branch targeted and the intended muscle group, the block technique, the timing of reassessment, and the main changes observed in passive ROM, active movement, and goal‐related function. A uniform anesthetic dose or a single threshold defining a “positive” response is not required.

Interpretation should focus on clinically meaningful changes. These include reduction in abnormal posture or passive resistance; improvement or persistence of passive restriction; changes in the direction, amplitude, or selectivity of active movement; emergence of underlying weakness or muscle force imbalance; and changes in goal‐related tasks such as grasp and release, foot placement, gait, hygiene, or dressing. When needed, DNB findings should be interpreted together with clinical examination, gait analysis, or electrophysiologic assessment.

When DNB is used before peripheral nerve surgery, the immediate response should not be translated directly into an expected long‐term surgical outcome. Persistent fixed restriction, inadequate voluntary recruitment, or muscle force imbalance after the block may indicate that another component remains functionally important and that the treatment plan should be reconsidered or combined with other reconstructive approaches.

Recommendation 4: In patients being considered for selective or hyperselective peripheral nerve surgery, DNB may be considered when the contribution of dynamic abnormal neural drive is uncertain, the principal contributing muscle group cannot be identified confidently, or the functional consequences of reducing neural drive need to be assessed before surgery. DNB is most useful when its findings are expected to influence subsequent treatment decisions. (Evidence support: limited.) It should be used as a decision‐support tool rather than as a stand‐alone predictor of surgical benefit. Direct supporting evidence is derived mainly from lower‐limb studies, and application to the upper limb should therefore be interpreted with caution.

Expert Consensus Statement 6: The principal role of DNB is to provide a reversible clinical test that helps clarify the contribution of dynamic abnormal neural drive and the functional consequences of reducing activity in the targeted muscles.

Expert Consensus Statement 7: Immediate changes after DNB do not reliably predict long‐term strength, joint ROM, or active function after selective or hyperselective peripheral nerve surgery and should not be used as the sole basis for surgical decision‐making.

### 6.2 Timing and procedural considerations for DNB

DNB does not need to be deferred until the chronic stage after stroke. It may be considered whenever there is a specific clinical question, and the findings are expected to influence subsequent management; no uniform minimum post‐stroke interval has been established.^4^, ^10^ When the contributing pathophysiologic components are already sufficiently clear, and DNB is unlikely to change treatment decisions, routine use is unnecessary. Procedural safety should follow established precautions for local anesthetic administration and peripheral nerve block. DNB should be avoided in the presence of infection at the intended injection site, known allergy to the planned local anesthetic, or an unacceptable risk of bleeding.

## 7 SELECTIVE/HYPERSELECTIVE PERIPHERAL NERVE SURGERY

### 7.1 Treatment goals and patient selection

Selective or hyperselective peripheral nerve surgery aims to achieve a sustained reduction in abnormal neural drive to specific target muscles. The intended benefits may include improvement in abnormal posture, pain, hygiene or dressing, walking, and caregiver burden. Because these procedures are irreversible, preoperative assessment should go beyond grading the severity of spasticity. It should establish whether abnormal neural drive to the target muscles is an important contributor to the patient's current functional problem and whether the expected benefit outweighs the potential loss of useful muscle activity. International expert consensus on surgery for adult upper‐limb spasticity likewise emphasizes clear treatment goals, careful patient selection, and standardized outcome assessment.^43^

Recommendation 5: Selective or hyperselective peripheral nerve surgery may be considered in patients with persistent, focal, goal‐relevant dynamic abnormal muscle overactivity despite appropriate rehabilitation and nonsurgical management, when comprehensive assessment indicates that sustained reduction of neural drive to the target muscle is likely to improve function, posture, symptoms, or care with acceptable functional trade‐offs.

(Evidence support: substantial for reduction of target spasticity; limited for improvement in active function.)

The relevant motor pattern and treatment goals should be sufficiently stable to support surgical planning and postoperative rehabilitation. Current randomized evidence is derived mainly from patients with refractory spasticity, including those with MAS grades 3–4 and an inadequate response to maximally tolerated baclofen. Extrapolation to patients with milder spasticity or different prior treatment pathways should therefore be cautious.

### 7.2 Randomized evidence in refractory post‐stroke spasticity

In 2026, Bajaj et al. reported a prospective randomized controlled trial involving 50 patients with post‐stroke spasticity graded MAS 3–4 who had responded inadequately to maximally tolerated baclofen. Participants were randomized 1:1 to hyperselective peripheral neurectomy plus rehabilitation or continued medical therapy plus rehabilitation.^12^ At 12 months, the reduction in MAS was 1.36 points greater in the surgical group than in the control group (95% CI, 0.97–1.74; *p* < 0.001), and 96% of surgical patients improved by at least one MAS grade compared with 8% of controls.

Functional independence improved from baseline within the surgical group, but the between‐group difference was not statistically significant. No significant between‐group differences were observed in muscle strength or sensory outcomes. Five patients in the surgical group developed transient neuropathic pain, which resolved within 2–3 months with medical treatment.

This trial provides direct randomized evidence that hyperselective peripheral neurectomy can reduce severe refractory post‐stroke spasticity. However, the functional findings were less definitive, underscoring that a reduction in spasticity should not be assumed to produce a corresponding improvement in functional independence.

### 7.3 Upper‐limb HSN and evidence on long‐term outcomes

A 2026 systematic review by Jerome et al. included 10 studies involving 260 adults with upper‐limb spasticity due to stroke, traumatic brain injury, cerebral palsy, multiple sclerosis, or spinal cord injury. Across six studies suitable for pooling, MAS decreased by a mean of 1.64 points (95% CI, 1.36–1.92).^13^ Most of the included studies, however, were noncomparative case series and were classified as level IV evidence. A second systematic review published in the same year included nine studies covering 253 HSN procedures and similarly reported reductions in spasticity, with strength and ROM generally preserved or slightly improved.^14^ Interpretation was limited by substantial heterogeneity in patient populations, surgical techniques, follow‐up duration, and outcome measures. Available studies suggest that upper‐limb HSN can provide a sustained reduction in spasticity. Evidence for improvement in active function, particularly in patients with stroke, remains limited.

Expert Consensus Statement 8: The principal goal of selective or hyperselective peripheral nerve surgery is sustained reduction of abnormal neural drive to the target muscles, with improvement in goal‐relevant posture, symptoms, function, or care.

Expert Consensus Statement 9: Reduction in muscle tone after selective or hyperselective peripheral nerve surgery should not be interpreted as evidence of recovery in active movement, activity performance, or functional independence.

### 7.4 Typical lower‐limb application: Post‐stroke spastic equinovarus foot

Post‐stroke spastic equinovarus foot usually reflects more than spasticity alone. Abnormal neural drive to the calf muscles, shortening of the triceps surae–Achilles tendon complex, and weakness or imbalance of the ankle dorsiflexors and evertors may contribute separately or in combination.^4^, ^5^ In a 2013 assessor‐blinded RCT, Bollens et al. enrolled 16 patients with chronic post‐stroke spastic equinovarus foot and compared selective tibial neurotomy with botulinum toxin treatment.^15^ Tibial neurotomy produced a greater reduction in ankle stiffness, but no significant between‐group differences were demonstrated in activity, participation, or quality of life. A 2024 systematic review by Ploegmakers et al. included 25 nonrandomized or uncontrolled studies and one RCT and found that selective neurotomy was associated with improvements in lower‐limb muscle tone, pain, passive ankle ROM, and walking speed.^44^ The evidence, however, was derived largely from uncontrolled case series, and the included populations covered a range of upper motor neuron syndromes rather than stroke alone.

### 7.5 Timing and situations requiring caution or avoidance

Selective or hyperselective peripheral nerve surgery is generally not considered an initial treatment in the early post‐stroke period. Surgical evaluation is more appropriate after an adequate period of rehabilitation and clinically indicated reversible treatment, when the pattern of abnormal muscle overactivity and its effect on the target function have become sufficiently stable. Clinical assessment, supplemented by DNB when needed, should support that abnormal neural drive to the target muscles is an important contributor to the problem and that reducing it is likely to provide meaningful benefit.

Reviews of reconstructive surgery in adults after stroke often cite approximately 6 months after brain injury as a practical reference point for considering irreversible reconstruction.^39^, ^40^ This interval is based largely on clinical experience and review‐level recommendations rather than a prospectively validated surgical threshold and should not replace individualized clinical judgment.

Peripheral nerve surgery alone may provide limited benefit when the predominant limitation is fixed contracture, substantial joint structural restriction, or severe impairment of voluntary motor recruitment. Particular caution is also required when increased muscle tone contributes to a useful compensatory function, the motor pattern remains unstable, perioperative risk is unacceptable, or the patient is unable to participate in postoperative rehabilitation.

## 8 FIXED STRUCTURAL CHANGES AND TENDON/SOFT‐TISSUE FUNCTIONAL RECONSTRUCTION

### 8.1 Identification of fixed structural changes

Fixed structural restriction becomes clinically important when a persistent mechanical limitation remains after dynamic neural factors have been considered or reduced. When such restriction is a major barrier to the patient's functional goal, further reduction of abnormal neural drive alone is unlikely to restore sufficient passive motion, and the need for structural reconstruction should be considered.

### 8.2 Tendon lengthening and soft‐tissue release

Tendon lengthening and muscle or fascial release are used primarily to address established shortening or contracture of the muscle‐tendon unit and other soft tissues. Treatment goals may include improving passive ROM, limb posture, hygiene, pain, footwear or orthosis fit, and the mechanical conditions needed for subsequent active rehabilitation.

A 2024 systematic review by Hurtado‐Olmo et al. included 22 studies of surgery for post‐stroke spastic hand.^16^ The evidence was predominantly of low or very low quality, with most studies consisting of case series or retrospective designs. Available findings suggest that selected patients may benefit in passive ROM, posture, hygiene, and some functional goals, but comparative evidence remains limited and the relative effectiveness of individual procedures is uncertain.

Recommendation 6: In patients with established muscle‐tendon shortening or soft‐tissue contracture that meaningfully limits function, hygiene, pain control, footwear or orthosis fit, limb posture, or subsequent rehabilitation, tendon lengthening, muscle or fascial release, or another appropriate soft‐tissue procedure may be considered when the restriction cannot be adequately corrected by reducing dynamic abnormal neural drive alone.

(Evidence support: limited.)

### 8.3 Tendon transfer and dynamic reconstruction

The purpose of tendon transfer is not simply to increase strength or to treat centrally mediated spasticity. When useful voluntary muscle power is preserved, tendon transfer redirects the insertion and line of pull of a donor muscle so that its existing force can contribute to a desired movement. A systematic scoping review by Chan et al. included 61 studies involving 2293 adults with equinovarus foot after acquired brain injury and found substantial variation in preoperative assessment and surgical selection criteria, with no validated uniform indications.^18^ Current evidence therefore does not support determining candidacy on the basis of a single numeric threshold, such as an MRC strength grade or FMA score.

Expert Consensus Statement 10: Selection for tendon transfer should consider the consistency of voluntary donor‐muscle recruitment, muscle strength, activation pattern, and direction of action, together with passive ROM of the target joint, antagonist activity, and the patient's ability to participate in postoperative motor relearning. A single MRC grade, FMA score, or other numeric threshold should not be used alone to determine candidacy.

### 8.4 Timing and situations requiring caution in structural or dynamic reconstruction

The need for tendon lengthening or soft‐tissue release should be determined primarily by the presence and functional importance of fixed structural restriction rather than by time since stroke alone. Structural reconstruction may be considered when a clinically meaningful mechanical restriction persists during slow passive examination or after reduction of dynamic abnormal neural drive and continues to interfere with the patient's functional goal. When the limitation is predominantly dynamic, and no clear fixed shortening is present, premature or excessive lengthening may reduce useful muscle force.

Tendon transfer requires a donor muscle that can be recruited voluntarily and consistently, adequate passive ROM at the target joint, manageable antagonist forces, and sufficient capacity to complete postoperative immobilization and rehabilitation.^18^, ^23^ Particular caution is required when donor‐muscle recruitment is unstable, fixed joint restriction remains uncorrected, the movement pattern is still changing substantially, or the patient is unable to participate in postoperative motor relearning.

## 9 MIXED PATHOPHYSIOLOGIC COMPONENTS AND INDIVIDUALIZED COMBINED RECONSTRUCTION

### 9.1 Basic principles of individualized combined reconstruction

Patients with post‐stroke spastic hemiplegia often have more than one pathophysiologic component contributing to the same functional problem. Dynamic abnormal muscle overactivity, fixed structural restriction, and muscle force imbalance may coexist and may each require a different treatment approach. Combined reconstruction should therefore be based on clearly identified treatment targets rather than on routine use of multiple procedures.

### 9.2 Current evidence for individualized combined reconstruction

In 2018, Deltombe et al. reported a prospective, single‐blind, single‐group before‐and‐after study involving 18 patients with post‐stroke spastic equinovarus foot. According to the pathophysiologic components identified in each patient, treatment included selective tibial neurotomy, Achilles tendon lengthening, tibialis anterior tendon transfer, or a combination of these procedures. At 1 year, improvements were observed in GAS, spasticity, pain, ankle ROM, walking speed, and selected activity and participation outcomes.^19^ These findings support the feasibility of tailoring neuro‐orthopedic reconstruction to individual functional goals and pathophysiologic findings. However, the study does not establish the superiority of any fixed combination of procedures or determine the independent contribution of each surgical component.

### 9.3 Individualization of combined procedures and treatment sequence

Current evidence does not establish that any fixed combination of peripheral nerve surgery, soft‐tissue reconstruction, and tendon transfer is superior to an individualized approach. Evidence is also insufficient to support a uniform preference for either single‐stage or staged reconstruction.

When several components materially limit the same functional goal, they may be addressed during the same operative episode when clinically appropriate. In other cases, treatment may proceed in stages, with the predominant limiting component addressed first and residual problems reassessed before further reconstruction is considered.

Recommendation 7: In patients with coexisting goal‐relevant dynamic abnormal muscle overactivity, fixed structural restriction, and/or muscle force imbalance, individualized combined reconstruction may be considered when more than one component materially limits the established functional goal. The choice and timing of procedures should be guided by the predominant pathophysiologic components, residual voluntary motor capacity, tissue and joint conditions, surgical complexity, and postoperative rehabilitation requirements.

(Evidence support: very limited.)

Direct evidence for combined reconstruction is derived mainly from small observational studies and does not demonstrate the superiority of any fixed combination. Peripheral nerve surgery, tendon or soft‐tissue reconstruction, and dynamic reconstruction may therefore be combined when their respective treatment targets are present, but should not be applied as a predetermined package.

Expert Consensus Statement 11: Current evidence does not establish that any fixed combination of peripheral nerve surgery, tendon lengthening or soft‐tissue release, and tendon transfer is superior to an individualized reconstructive strategy. This consensus therefore does not prescribe a uniform single‐stage or staged combined procedure.

The same principle applies to BCI, selective or hyperselective peripheral nerve surgery, and tendon transfer. These interventions address different pathophysiologic components and should not be predefined as a standard combination. Each should be selected according to the patient's functional goal and predominant limiting factor, with reassessment after treatment to determine whether additional targets remain.

## 10 CLINICAL DECISION PATHWAY FOR NEURO‐MUSCULOSKELETAL FUNCTIONAL RECONSTRUCTION

Neuro‐musculoskeletal functional reconstruction for post‐stroke spastic hemiplegia should begin with the patient's functional goal rather than with the selection of a particular intervention. Once the goal is defined, the relative contributions of the four pathophysiologic dimensions should be assessed to identify the predominant limiting component and the principal treatment target. This consensus therefore proposes a clinical pathway of “patient goal–pathophysiologic component–treatment target–functional reassessment,” without prescribing a fixed combination or sequence of interventions.

When impaired central voluntary motor control is the predominant limitation and further active recovery is a treatment goal, task‐specific motor training should remain the foundation of rehabilitation. BCI, FES, robotics, or other feedback‐based approaches may be added in appropriately selected patients. The 2024 Department of Veterans Affairs/Department of Defense (VA/DoD) Clinical Practice Guideline for Management of Stroke Rehabilitation likewise emphasizes task‐specific training as a core strategy for motor recovery.^45^

For dynamic abnormal muscle overactivity, fixed structural restriction, or muscle force imbalance, treatment should be directed at the corresponding pathophysiologic target. The relevant assessment tools, interventions, and clinical boundaries are described in Sections 6–9. If more than one component contributes meaningfully to the same functional problem, treatment may be combined or staged according to the individualized principles outlined above rather than a predetermined regimen. Functional reassessment should follow each stage of treatment. Clinicians should determine whether the intended goal has been achieved or meaningfully improved, which limitations remain, and whether an additional intervention is likely to provide further clinically relevant benefit.

The integrated decision pathway is summarized in Figure 1. At each stage, subsequent decisions should be guided by the patient's current functional priorities, residual pathophysiologic limitations, treatment reversibility, tissue and joint conditions, and the feasibility of further rehabilitation.

**FIGURE 1:**
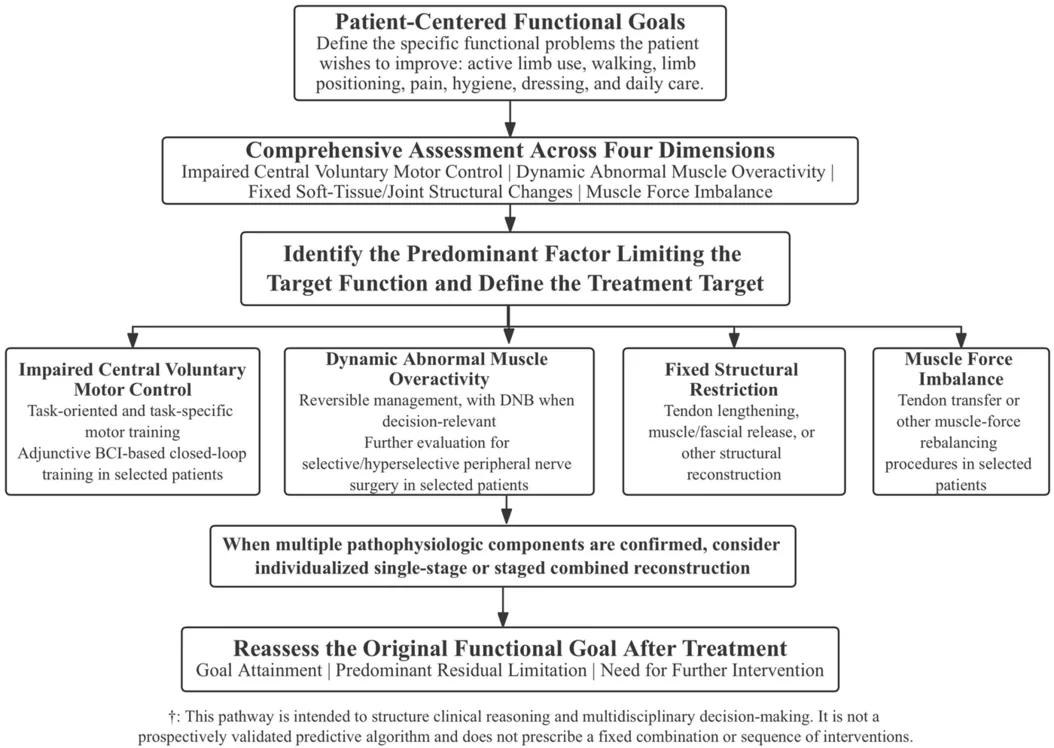
Clinical decision pathway for neuro‐musculoskeletal functional reconstruction in post‐stroke spastic hemiplegia.

## 11 MOTOR RELEARNING AFTER PERIPHERAL FUNCTIONAL RECONSTRUCTION

### 11.1 Recovery of active function after peripheral reconstruction

Peripheral nerve surgery, soft‐tissue release, and tendon transfer can modify abnormal neural drive, joint mechanics, or the direction of muscle action, but these changes do not by themselves restore active function. Following selective or hyperselective peripheral nerve surgery, voluntary movement must adapt to the altered balance between agonist and antagonist muscles. Correction of fixed structural restriction may increase available joint ROM, but this new range must be incorporated into purposeful movement through active control and task‐specific training. After tendon transfer, the patient must learn to recruit the donor muscle for its new mechanical function.

### 11.2 Postoperative motor training according to the specific procedure and tissue healing

Evidence from stroke rehabilitation supports task‐specific training for improving motor function, gait, and activities of daily living,^45^ However, high‐quality evidence is lacking to define a single start time, training dose, or loading protocol after selective peripheral nerve surgery, tendon lengthening, or tendon transfer. Postoperative rehabilitation should therefore be adapted to the procedure performed, tissue‐healing stage, immobilization requirements, residual voluntary motor capacity, and the patient's functional goals.

After tendon transfer in particular, progression from active recruitment to resistance training and increased loading should be coordinated by the surgical and rehabilitation teams according to tissue healing and the immobilization strategy. Electrical stimulation may be incorporated when appropriate, but postoperative progression should not follow a uniform timetable irrespective of the procedure and healing status.

Expert Consensus Statement 12: After peripheral nerve or tendon and soft‐tissue functional reconstruction, staged motor retraining should be tailored to the procedure and tissue‐healing requirements. The aim is to translate postoperative neural, structural, or biomechanical changes into selective voluntary movement and goal‐relevant activity. Surgical reconstruction itself should not be regarded as the endpoint of functional reconstruction.

### 11.3 Role of BCI after peripheral functional reconstruction

BCI may have a potential role in supporting motor relearning after peripheral functional reconstruction, particularly after tendon transfer, where feedback linked to motor intention could theoretically assist learning of the donor muscle's new function. Direct clinical evidence, however, is currently lacking. Most BCI studies have involved general post‐stroke rehabilitation populations, and no adequate evidence shows that adding BCI after selective or hyperselective peripheral nerve surgery or tendon reconstruction is superior to standard postoperative motor training, accelerates motor relearning, or improves active function and patient‐centered outcomes.

Expert Consensus Statement 13: There is currently insufficient direct clinical evidence to support routine BCI training after peripheral nerve or tendon functional reconstruction. BCI should therefore not be considered a standard component of postoperative rehabilitation at present, and its potential role should be evaluated in prospective clinical studies.

The core evidence, evidence‐supported conclusions, and main limitations for the major clinical questions addressed above are summarized in Table 4.

**TABLE 4:** Summary of core evidence for the major clinical questions.

Clinical question	Core evidence	Evidence‐supported conclusion	Main limitations
Multidimensional functional assessment	Bandela et al., 2026^3^; Deltombe et al., 2017^4^; Salga et al., 2023.^5^ Existing statements/consensus documents emphasize integrated interpretation of abnormal muscle activity, voluntary movement, structural factors, and muscle force.	[Support: substantial] Supports multidimensional assessment as the basis for clinical reasoning; the four‐dimensional integration used here is a consensus framework.	The four‐dimensional framework itself has not been prospectively validated as a predictive model.
Overall efficacy of BCI	Qin et al., 2026^7^: 43 studies, 1628 participants; compared with usual care, only a small benefit for upper‐limb function in 10 studies/611 participants, and no consistent advantage over sham BCI in 9 upper‐limb studies/279 participants. Li et al., 2025^8^: 21 RCTs, 886 participants; between‐group FMA‐UE mean difference = 3.69 (95% CI, 2.41–4.96), *I* ^2^ = 70%.	[Support: limited] Upper‐limb motor function may improve modestly; BCI may be used as an adjunct to standard rehabilitation.	Overall certainty is low to very low; heterogeneity is substantial; effects on activities of daily living, long‐term benefit, and the specific treatment effect remain uncertain.
BCI‐FES	Ren et al., 2024^9^: 10 RCTs, 290 participants; SMD for upper‐limb motor function = 0.50 (95% CI, 0.26–0.73).	[Support: limited] May be used as one modality of closed‐loop upper‐limb motor training.	Small studies; inconsistent paradigms, stimulation parameters, and training dose; limited long‐term evidence.
DNB	Archibold et al., 2026^11^: 9 studies, including 5 on neurotomy and 4 on botulinum toxin; all addressed the lower limb.	[Support: limited] May be used for reversible validation of pathophysiologic components and as an adjunct to decision‐making for selective/hyperselective peripheral nerve surgery**; direct supporting evidence is predominantly lower‐limb**.	Direct upper‐limb evidence is sparse; prediction of long‐term strength, ROM, and active function is limited; block protocols are heterogeneous.
Peripheral nerve surgery	Bajaj et al., 2026^12^: RCT, *n* = 50; between‐group MAS difference at 12 months 1.36 (95% CI, 0.97–1.74), ≥1‐grade improvement 96% versus 8%; no significant between‐group difference in functional independence; 5 cases of transient neuropathic pain. Jerome et al., 2026^13^: 10 studies/260 participants; pooled MAS improvement across 6 studies 1.64 (95% CI, 1.36–1.92).	[Support: substantial for spasticity reduction; limited for active function] Carefully selected patients with refractory disease may achieve sustained reduction of target spasticity.	The RCT was single‐center and small, with relatively severe patients; systematic reviews are dominated by case series and mixed etiologies, with insufficient evidence for active function/independence.
Selective tibial neurotomy	Bollens et al., 2013^15^: chronic stroke RCT, *n* = 16; Ploegmakers et al., 2024^44^: 25 nonrandomized/uncontrolled studies + 1 RCT.	[Support: limited] Can reduce focal lower‐limb spasticity and may improve some ROM, pain, and walking measures.	Direct RCT sample is very small; much of the evidence comes from case series; systematic review includes multiple upper motor neuron etiologies.
Fixed contracture/upper‐limb soft‐tissue surgery	Hurtado‐Olmo et al., 2024^16^: 22 studies; 21 (95.45%) rated low or very low quality by GRADE.	[Support: limited] Selected patients may improve posture, hygiene, pain, ROM, and some goals.	Evidence is dominated by observational case series; comparative studies are limited; procedures and patient goals are heterogeneous.
Tendon‐transfer selection	Chan et al., 2023^18^: 61 studies, 2293 adults with equinovarus foot after brain injury; marked inconsistency in preoperative assessment and procedure‐selection criteria.	[Support: limited] Supports integrated assessment of donor muscle, ROM, antagonists, and dynamic function; does not support a uniform single numeric threshold.	Populations, procedures, and preoperative assessments are highly heterogeneous; no validated selection model exists.
Individualized combined reconstruction	Deltombe et al., 2018^19^: 18 patients, single‐group pre‐post study, 1‐year follow‐up; neurotomy, Achilles tendon lengthening, and/or tendon transfer selected according to pathophysiologic composition.	[Support: very limited] Supports the clinical feasibility of individualized combinations based on patient goals and pathophysiologic components; does not establish superiority of combined over single procedures.	No control group and small sample; cannot show that a fixed combination is superior to a single procedure or isolate the independent effect of each surgical component.
Postoperative BCI	There are currently no direct comparative studies of added BCI training after peripheral nerve or tendon functional reconstruction.	[Support: no direct evidence] Mechanistically plausible and appropriate as a research question.	General post‐stroke BCI evidence cannot be extrapolated to make BCI a standard postoperative treatment.

*Note*: “Level of support” in this table is a simplified internal evidence judgment used in this consensus to indicate how strongly current evidence supports the corresponding clinical conclusion. It is not equivalent to GRADE certainty of evidence.

Abbreviations: BCI, brain‐computer interface; DNB, diagnostic motor nerve block; FES, functional electrical stimulation; FMA‐UE, Fugl‐Meyer Assessment–Upper Extremity; GRADE, Grading of Recommendations Assessment, Development and Evaluation; ROM, range of motion; SMD, standardized mean difference.

## 12 OUTCOME ASSESSMENT AND FOLLOW‐UP

### 12.1 Outcome assessment based on the treatment goal

Neuro‐musculoskeletal functional reconstruction after stroke addresses different pathophysiologic components and treatment goals, and no single outcome measure is appropriate for every patient or intervention. Outcome assessment should therefore reflect the goal established before treatment. For a patient whose primary goal is improved hand hygiene, pain relief, or easier dressing, treatment may still provide meaningful benefit even if active grasp does not improve.

### 12.2 Limitations of MAS in assessing outcomes of functional reconstruction

The MAS may be used to document changes in resistance to passive movement, but it does not adequately represent the broader functional effects of reconstruction. Changes in MAS should therefore be interpreted alongside active movement, activity, participation, and goal‐related outcomes. Randomized studies of peripheral nerve surgery have shown that muscle tone may improve substantially without a corresponding improvement in activity, participation, or functional independence.^12^, ^15^ Similarly, BCI studies have reported improvements in motor impairment, while effects on activities of daily living remain uncertain.^7^

### 12.3 ICF‐oriented multidimensional outcome assessment

This consensus recommends a multidimensional approach informed by the International Classification of Functioning, Disability and Health (ICF). Outcome measures should be selected according to the treatment goal and, where relevant, cover body functions and structures, activity and participation, and patient‐centered goals. GAS may be used to capture individualized goals. In the prospective Upper Limb International Spasticity Study II, which included 456 patients with post‐stroke upper‐limb spasticity, 79.6% achieved or exceeded their primary predefined goal; these goals included passive function, active function, pain, and other clinically relevant domains.^33^, ^34^

For interventions such as BCI, mechanistic or neurophysiologic measures, including ERD/ERS, MRCP, and classification accuracy, may provide useful supplementary information. They should not, however, replace measures of clinical function or patient‐centered benefit.

After surgical reconstruction for post‐stroke spastic equinovarus foot, gait assessment should likewise combine the patient's functional goals with objective measures where appropriate. A 2022 review by de l' Escalopier et al. included 33 studies and found considerable variation in postoperative assessment: 11 studies used instrumented gait analysis, and only 6 combined instrumented, clinical, and functional evaluation.^46^ These findings support the use of reliable quantitative gait measures alongside clinical and patient‐centered outcomes.

Expert Consensus Statement 14: Outcomes of neuro‐musculoskeletal functional reconstruction after stroke should not be judged solely by MAS or another single measure of muscle tone. Assessment should be aligned with the treatment goal and, where appropriate, include body functions and structures, activity and participation, and patient‐centered outcomes.

Expert Consensus Statement 15: Neurophysiologic and mechanistic measures, including BCI classification accuracy, ERD/ERS, MRCP, and dynamic EMG, may be used as supplementary outcomes but should not replace measures of active function, activity, participation, or patient‐centered benefit.

Suggested outcome measures across these assessment domains are summarized in Table 5.

**TABLE 5:** Suggested minimum reporting set and optional outcome measures for neuro‐musculoskeletal functional reconstruction.

Assessment domain	Optional upper‐limb measures	Optional lower‐limb measures
Minimum reporting set (all interventions)	Predefined patient‐centered goal; target‐region active and passive ROM; at least one goal‐relevant active movement or activity measure; treatment‐related adverse events/complications; need for further intervention; use the same measures at baseline and prespecified follow‐up whenever feasible.	Predefined patient‐centered goal; target‐region active and passive ROM; at least one goal‐relevant active movement or activity measure; treatment‐related adverse events/complications; need for further intervention; use the same measures at baseline and prespecified follow‐up whenever feasible.
Body functions and structures	Active/passive ROM, MAS/Tardieu, MRC, pain; dynamic EMG when needed	Active/passive ROM, MAS/Tardieu, MRC, pain; dynamic EMG/gait analysis when needed
Active movement	FMA‐UE, grasp‐and‐release ability	FMA‐LE, selective ankle/knee control
Activity	ARAT, Box and Block Test, and other goal‐related task measures	10‐m walk test, FAC, 6‐min walk test, and other relevant measures
Patient‐centered goals	GAS, hygiene, dressing, pain, caregiving difficulty, etc.	GAS, footwear fit, dependence on assistive devices, falls, and goal‐related walking problems, etc.
Research measures	ERD/ERS, MRCP, BCI classification accuracy, dynamic EMG, etc.	Selected according to the research question
Safety and further intervention	Procedure/device‐related adverse events, new weakness or sensory symptoms, wound/tissue‐healing problems when relevant, reoperation or additional treatment.	Procedure/device‐related adverse events, new weakness or sensory symptoms, wound/tissue‐healing problems when relevant, falls or gait deterioration, reoperation or additional treatment.

Abbreviations: ARAT, Action Research Arm Test; EMG, electromyography; ERD/ERS, event‐related desynchronization/synchronization; FAC, Functional Ambulation Category; FMA‐UE/FMA‐LE, Fugl‐Meyer Assessment–Upper/Lower Extremity; GAS, Goal Attainment Scaling; MAS, Modified Ashworth Scale; MRC, Medical Research Council Scale; MRCP, movement‐related cortical potential; ROM, range of motion.

The minimum reporting items in Table 5 are intended to improve comparability across studies and clinical datasets. Additional measures should be selected according to the treatment goal, body region, and research question, with the same goal‐relevant measures used at baseline and follow‐up whenever feasible. This framework is intended to guide multidimensional outcome reporting and should not be regarded as a formally developed core outcome set.

### 12.4 Long‐term follow‐up

Long‐term follow‐up after surgery should extend beyond wound healing, complications, and recurrence of spasticity. It should determine whether the neural, structural, or biomechanical changes achieved by reconstruction translate into sustained functional benefit and whether important residual or new limitations have emerged.

Follow‐up should be guided by the original treatment goals and may include goal attainment, active and passive ROM, target and antagonist muscle function, abnormal muscle overactivity, pain, task performance or walking ability, use of assistive devices, patient‐ or caregiver‐perceived benefit, treatment‐related adverse events, and the need for further reconstruction.

## 13 LIMITATIONS OF THE CURRENT EVIDENCE AND FUTURE RESEARCH DIRECTIONS

The strength of evidence varies across the interventions addressed in this consensus. BCI‐assisted training has been evaluated in multiple RCTs and systematic reviews, but heterogeneity in training paradigms, dose, comparators, and outcome measures remains substantial, and effects on activities of daily living and long‐term function are uncertain.^7^, ^8^, ^9^ Randomized evidence supports reduction of target spasticity after peripheral nerve surgery in selected patients with stroke, although evidence for improvement in active movement, activity, and participation remains limited.^12^, ^15^ Evidence for tendon lengthening, soft‐tissue release, and tendon transfer after stroke remains predominantly observational.^16^, ^17^, ^18^, ^19^ Evidence is also insufficient to establish the added clinical value of SWE or emerging interventions such as percutaneous cryoneurolysis within the core decision pathway.^29^, ^30^, ^31^, ^32^

This consensus also has methodological limitations. Primary database searches were limited to PubMed and CNKI, the historical CNKI search record could not be fully reconstructed, and the original item‐level numerical voting export was unavailable during final manuscript preparation. The panel was multidisciplinary but was weighted toward the initiating center, and patients and caregivers were not directly involved. These limitations should be addressed in future updates through broader evidence retrieval, improved data archiving, wider external representation, and structured patient and caregiver participation.

Patient selection and outcome assessment are also inconsistent across existing studies. Many reports rely heavily on MAS scores, abnormal posture, or local deformity, with limited characterization of the underlying pathophysiologic components. Outcomes frequently emphasize muscle tone, ROM, or local deformity, whereas active function, activity and participation, and patient‐centered goals are reported less consistently. Evidence comparing treatment sequences is particularly sparse. The four‐dimensional framework and clinical pathway proposed here should therefore be regarded as tools for organizing clinical reasoning rather than as prospectively validated predictive models.

Future Research Priority 1: Multicenter prospective studies should evaluate motor retraining after selective or hyperselective peripheral nerve surgery, including whether closed‐loop approaches such as BCI provide additional benefit in active function, activity and participation, goal attainment, and long‐term outcomes.

Future Research Priority 2: Prospective studies after tendon transfer should characterize postoperative motor relearning and determine whether BCI‐assisted feedback can improve recruitment of the transferred muscle and goal‐related function.

Future Research Priority 3: Multicenter prospective registries should standardize documentation of pathophysiologic components, DNB findings, treatment selection, surgical procedures, rehabilitation, and long‐term outcomes, and may provide a basis for development of a future core outcome set.

## 14 CONCLUSION

Persistent motor dysfunction in post‐stroke spastic hemiplegia rarely results from abnormal muscle tone alone. Impaired voluntary motor control, abnormal muscle overactivity, fixed structural restriction, and muscle force imbalance often coexist, and their relative importance may change over time. Functional reconstruction should therefore start from the patient's goals and seek to identify the main factor limiting the desired function and the corresponding treatment target.

Treatment should then be tailored to that target. Task‐specific motor training remains central when impaired voluntary motor control is the main problem, and BCI‐based closed‐loop training may provide an additional option for selected patients. Persistent focal muscle overactivity driven predominantly by abnormal neural input may be treated with selective or hyperselective peripheral nerve surgery in appropriately assessed patients. Fixed restriction may require tendon or soft‐tissue procedures, while tendon transfer or other dynamic reconstruction may be useful when muscle force imbalance is a major limitation. Many patients have more than one of these problems, and treatment may therefore need to be staged or combined.

Surgery changes the conditions under which movement occurs; it does not by itself restore active function. Postoperative motor retraining and repeated functional assessment are therefore essential. Success should be judged by whether treatment achieves goals that matter to the patient, rather than by a change in muscle tone alone.

A standard combination or fixed sequence of BCI, peripheral nerve surgery, and tendon or soft‐tissue reconstruction cannot presently be recommended. The pathway of “patient goal‐pathophysiologic component‐treatment target‐functional reassessment” is intended to support individualized clinical decision‐making. Further prospective multicenter studies are needed to improve patient selection, clarify treatment sequencing, and establish long‐term functional outcomes.

## AUTHOR CONTRIBUTIONS

Peng Wen conceived and designed the consensus, conducted the literature review, and drafted the initial manuscript. Yihe Wang contributed to consensus development, expert discussions, and manuscript revision. Zhongzheng He, Zhizhong Zhu, and Chunbing Chen participated in evidence evaluation, provided professional input, and contributed to manuscript revision. Kaifeng Wu, Xiaohong Fu, Guoguang Zhao, and Liulin Xiong supervised the consensus development, coordinated expert opinions, and critically revised the manuscript. All authors reviewed and approved the final version of the manuscript.

## MEMBERS OF THE CONSENSUS EXPERT PANEL (LISTED IN ALPHABETICAL ORDER BY SURNAME)

Chunbing Chen (Department of Radiology, The First People's Hospital of Zunyi [The Third Affiliated Hospital of Zunyi Medical University]); Denggui Cheng (Department of Neurosurgery, The First People's Hospital of Zunyi [The Third Affiliated Hospital of Zunyi Medical University]); Zhaojian Deng (Department of Neurosurgery, The First People's Hospital of Zunyi [The Third Affiliated Hospital of Zunyi Medical University]); Xiaohong Fu (Brain‐Computer Interface Medical Center, The First People's Hospital of Zunyi [The Third Affiliated Hospital of Zunyi Medical University]); Min Gong (Cerebrovascular Department, The First People's Hospital of Zunyi [The Third Affiliated Hospital of Zunyi Medical University]); Chong Han (Cerebrovascular Department, Affiliated Hospital of Zunyi Medical University); Zhongzheng He (Department of Neurosurgery, Tianjin Huanhu Hospital); Qijun Jiang (Department of Neurosurgery, The First People's Hospital of Zunyi [The Third Affiliated Hospital of Zunyi Medical University]); Hang Li (Cerebrovascular Department, The First People's Hospital of Zunyi [The Third Affiliated Hospital of Zunyi Medical University]); Jianping Li (Neurocritical Care Unit, The First People's Hospital of Zunyi [The Third Affiliated Hospital of Zunyi Medical University]); Shun Li (Department of Neurosurgery, Affiliated Hospital of North Sichuan Medical College); Yu Li (Brain‐Computer Interface Medical Center, The First People's Hospital of Zunyi [The Third Affiliated Hospital of Zunyi Medical University]); Minjie Luo (Department of Neurosurgery, Zhujiang Hospital, Southern Medical University); Chiyuan Ma (Department of Neurosurgery, General Hospital of Eastern Theater Command); Yongzhi Shan (Department of Neurosurgery, Xuanwu Hospital, Capital Medical University); Jun Wang (Department of Neurosurgery, Zhujiang Hospital, Southern Medical University); Nana Wang (Brain‐Computer Interface Medical Center, The First People's Hospital of Zunyi [The Third Affiliated Hospital of Zunyi Medical University]); Qinglan Wang (Brain‐Computer Interface Medical Center, The First People's Hospital of Zunyi [The Third Affiliated Hospital of Zunyi Medical University]); Yihe Wang (Department of Neurosurgery, Xuanwu Hospital, Capital Medical University); Ziheng Wang (Brain‐Computer Interface Medical Center, The First People's Hospital of Zunyi [The Third Affiliated Hospital of Zunyi Medical University]); Zhongying Wei (Brain‐Computer Interface Medical Center, The First People's Hospital of Zunyi [The Third Affiliated Hospital of Zunyi Medical University]); Peng Wen (Brain‐Computer Interface Medical Center, The First People's Hospital of Zunyi [The Third Affiliated Hospital of Zunyi Medical University]); Guojun Wu (Department of Neurology, Yutian County Hospital of Traditional Chinese Medicine); Kaifeng Wu (Department of Clinical Laboratory, The First People's Hospital of Zunyi [The Third Affiliated Hospital of Zunyi Medical University]); Bo Xiong (Department of Neurology, The First People's Hospital of Zunyi [The Third Affiliated Hospital of Zunyi Medical University]); Liulin Xiong (Brain‐Computer Interface Medical Center, The First People's Hospital of Zunyi [The Third Affiliated Hospital of Zunyi Medical University]); Jianguo Xu (Department of Neurosurgery, West China Hospital, Sichuan University); Minpeng Xu (Haihe Laboratory of Brain‐Computer Interaction and Human‐Machine Integration, Tianjin University); Kaihua Yang (Department of Neurosurgery, The First People's Hospital of Zunyi [The Third Affiliated Hospital of Zunyi Medical University]); Xi Yang (Brain‐Computer Interface Medical Center, The First People's Hospital of Zunyi [The Third Affiliated Hospital of Zunyi Medical University]); Fan Zhang (Department of Neurosurgery, West China Hospital, Sichuan University); Hongtian Zhang (Department of Neurosurgery, The Seventh Medical Center of PLA General Hospital); Jing Zhang (Brain‐Computer Interface Medical Center, The First People's Hospital of Zunyi [The Third Affiliated Hospital of Zunyi Medical University]); Shaofu Zhang (Cerebrovascular Department, The First People's Hospital of Zunyi [The Third Affiliated Hospital of Zunyi Medical University]); Guoguang Zhao (Department of Neurosurgery, Xuanwu Hospital, Capital Medical University); Hongxin Zhao (Cerebrovascular Department, Affiliated Hospital of Zunyi Medical University); Min Zhao (Department of Pharmacy, The First People's Hospital of Zunyi [The Third Affiliated Hospital of Zunyi Medical University]); Mingjiang Zhu (Brain‐Computer Interface Medical Center, The First People's Hospital of Zunyi [The Third Affiliated Hospital of Zunyi Medical University]); Zhizhong Zhu (Department of Rehabilitation Medicine, Tianjin Huanhu Hospital).

## CONFLICT OF INTEREST STATEMENT

The authors declare no conflicts of interest.

## ETHICS STATEMENT

As this study involved expert consensus and did not collect patient data, formal research ethics approval was not required.

## Supporting information

Supporting File 1.

## Data Availability

No individual patient‐level dataset was generated for this expert consensus. The supplementary materials provide details of the literature search strategy and evidence synthesis process. The final evidence base was derived from PubMed and CNKI searches, reference screening, and expert‐nominated literature relevant to the prespecified clinical questions.
