# Supplementary material for: Expert consensus on neuro‐musculoskeletal functional reconstruction for post‐stroke spastic hemiplegia
Source: Ibrain. 2026 Oct 27:10.1002/ibra.70032. Online ahead of print. doi: 10.1002/ibra.70032 (PMC13711417; doi:10.1002/ibra.70032)
Supplement: Supplementary file 1 [file IBRA-9999-0-s001.docx]

# SUPPLEMENTARY MATERIALS

# Expert Consensus on Neuro-Musculoskeletal Functional Reconstruction for Post-Stroke Spastic Hemiplegia

# Supplementary Methods S1. Consensus panel selection, composition, and patient involvement

The final consensus expert panel comprised 39 members from 11 institutions. Experts were purposively invited on the basis of substantive clinical or research experience relevant to post-stroke spastic motor dysfunction, rehabilitation medicine, neurology, peripheral nerve/functional neurosurgery, hand surgery/orthopedics, physical therapy, occupational therapy, clinical neurophysiology, BCI/neuroengineering, and related fields. The aim was multidisciplinary clinical and technical coverage rather than statistically proportional national representation.

The First People's Hospital of Zunyi served as the initiating and coordinating center and contributed 23 panel members. The remaining 16 members represented 10 external institutions. The larger contribution from the coordinating center reflects its responsibility for organization, evidence collation, coordination, and primary manuscript drafting. External experts were included to provide independent specialty and institutional perspectives.

Patients and caregivers were not directly involved in clinical-question formulation, evidence appraisal, drafting, or voting in this version of the consensus. The panel included clinicians with extensive direct experience caring for the target population; however, clinical experience does not substitute for patient representation. Future updates will seek structured patient and caregiver participation in question prioritization, interpretation of patient-important outcomes, and review of final recommendations.

**Supplementary Table S1. Institutional distribution of the consensus expert panel**

| Institution | Members, n | Interpretive note |
| --- | --- | --- |
| The First People's Hospital of Zunyi | 23 | Initiating/coordinating center; organization, evidence collation, coordination, and primary drafting. |
| Xuanwu Hospital, Capital Medical University | 3 | External institutional representation. |
| Affiliated Hospital of Zunyi Medical University | 2 | External institutional representation. |
| Tianjin Huanhu Hospital | 2 | External institutional representation. |
| West China Hospital, Sichuan University | 2 | External institutional representation. |
| Zhujiang Hospital, Southern Medical University | 2 | External institutional representation. |
| Affiliated Hospital of North Sichuan Medical College | 1 | External institutional representation. |
| General Hospital of Eastern Theater Command | 1 | External institutional representation. |
| Haihe Laboratory of Brain-Computer Interaction and Human-Machine Integration, Tianjin University | 1 | External institutional representation. |
| The Seventh Medical Center of PLA General Hospital | 1 | External institutional representation. |
| Yutian County Hospital of Traditional Chinese Medicine | 1 | External institutional representation. |

# Supplementary Methods S2. Literature search and evidence-identification audit

PubMed was the primary English-language database and CNKI the primary Chinese-language database. The archived PubMed search was run on August 30, 2026 using seven prespecified topic modules, each limited to publications from January 1, 2016 through August 30, 2026. The exact PubMed search strings are reproduced in Supplementary Appendix S1. The consensus was evidence-informed and did not perform an independent de novo systematic search in Embase, the Cochrane Library, Web of Science, Wanfang Data, or SinoMed.

The archived CNKI file is a union export rather than a preserved line-by-line query-history log. The exact historical CNKI search date and query syntax could not be verified from the archived materials available for this revision and were therefore not reconstructed post hoc. The exported records were audited directly, and Chinese-language concepts corresponding to stroke/post-stroke spastic motor dysfunction, BCI/FES, diagnostic motor nerve block, selective/hyperselective peripheral nerve surgery, tendon transfer/lengthening, soft-tissue release, contracture, rehabilitation, and motor relearning were used in the evidence-identification process.

**Supplementary Table S2. PubMed module-level search audit**

| Module | Search date | Publication window | Query hits | Rows in archived export |
| --- | --- | --- | --- | --- |
| Comprehensive assessment and pathophysiologic components | 2026-08-30 | 2016-01-01 to 2026-08-30 | 852 | 851 |
| Noninvasive BCI motor rehabilitation | 2026-08-30 | 2016-01-01 to 2026-08-30 | 891 | 891 |
| BCI-FES closed-loop training | 2026-08-30 | 2016-01-01 to 2026-08-30 | 132 | 132 |
| Diagnostic motor nerve block | 2026-08-30 | 2016-01-01 to 2026-08-30 | 12 | 12 |
| Selective/hyperselective peripheral nerve surgery | 2026-08-30 | 2016-01-01 to 2026-08-30 | 79 | 79 |
| Tendon and soft-tissue reconstruction | 2026-08-30 | 2016-01-01 to 2026-08-30 | 20 | 20 |
| Post-reconstruction motor relearning and rehabilitation | 2026-08-30 | 2016-01-01 to 2026-08-30 | 15 | 15 |

The PubMed query log recorded 2,001 module-level hits. The archived raw module export contains 2,000 rows. The one-record difference is confined to the comprehensive-assessment module (852 query hits versus 851 archived rows) and is reported transparently rather than imputed. After deduplication across modules by PMID, 1,827 unique PubMed records remained.

**Supplementary Table S3. Cross-source evidence-identification audit**

| Audit step | Count | Rule/interpretation |
| --- | --- | --- |
| PubMed module-level query hits | 2,001 | Sum of the seven archived PubMed query-log hit counts. |
| Rows in archived PubMed module export | 2,000 | One fewer than the query log because one module-level record was not present in the archived export. |
| Unique PubMed records | 1,827 | Deduplicated by PMID across the seven modules. |
| Rows in archived CNKI union export | 130 | All rows in the supplied CNKI union file. |
| Unique CNKI records | 117 | Deduplicated within CNKI using the archived DedupKey (DOI where available, otherwise normalized title). |
| PubMed-CNKI overlapping records | 20 | Matched on DOI and/or normalized exact title. |
| Unique electronic evidence pool | 1,924 | Unique PubMed + unique CNKI - cross-source overlap. |
| Final bibliography references directly represented in electronic pool | 20 | 19 matched PubMed; 2 matched CNKI; one reference was present in both. |
| Final bibliography references added by supplementary identification | 26 | Citation tracking and/or expert nomination followed by relevance appraisal. |
| Final bibliography | 46 | References cited in the submitted manuscript. |

The electronic evidence pool was not treated as a PRISMA-style de novo systematic review dataset. Screening and selection were directed by the prespecified clinical questions and evidence hierarchy, and the final bibliography intentionally emphasized evidence most directly needed to support the consensus statements, recommendations, definitions, and methodological framework.

Directly matched final references (reference numbers): 4, 7, 8, 9, 10, 12, 13, 14, 15, 17, 19, 21, 23, 29, 30, 32, 37, 39, 41, 44. Supplementarily identified final references (reference numbers): 1, 2, 3, 5, 6, 11, 16, 18, 20, 22, 24, 25, 26, 27, 28, 31, 33, 34, 35, 36, 38, 40, 42, 43, 45, 46.

# Supplementary Methods S3. Internal evidence-support framework

These categories are internal consensus descriptors and are not GRADE certainty categories.

| Category | Operational meaning | Typical evidence pattern | Implication for wording |
| --- | --- | --- | --- |
| Substantial | Convergent and clinically direct support from higher-level or comparative evidence without a major inconsistency that would materially change the conclusion. | Guideline/systematic review plus direct comparative evidence, or multiple concordant comparative studies. | May support 'recommend' or a clearly bounded action statement. |
| Limited | Direct clinical evidence exists, but important concerns remain regarding risk of bias, heterogeneity, indirectness, imprecision, sample size, or evidence volume. | Small RCT(s), low-certainty systematic-review evidence, or heterogeneous comparative/observational evidence. | Generally supports 'may be considered' with explicit implementation limits. |
| Very limited | Evidence is derived mainly from small observational studies, uncontrolled series, mixed etiologies, or substantially indirect evidence. | Case series, small single-group cohorts, or indirect mechanistic/clinical evidence. | Use cautious wording and avoid implying comparative superiority. |
| No direct evidence | No directly relevant clinical study addresses the specific proposed action. | Mechanistic plausibility, indirect evidence, or expert judgment only. | Prefer a consensus statement or research priority rather than routine recommendation. |

# Supplementary Methods S4. Consensus discussion, voting, and recoverability of numerical data

The final expert panel comprised 39 members. Draft items were prereviewed, discussed in a structured consensus meeting, and independently scored on a 9-point Likert scale. Scores of 1–3 indicated disagreement, 4–6 uncertainty or a need for revision, and 7–9 agreement. Consensus was prespecified as at least 80% of votes in the 7–9 range and less than 15% in the 1–3 range; an item with at least 80% agreement but 15% or more disagreement was considered to have substantive disagreement. Items that failed to reach consensus or underwent substantive revision after discussion were revised and resubmitted for a second vote. Items failing the prespecified criteria after the second vote were excluded from the final recommendations and consensus statements.

The original item-level electronic voting export was no longer recoverable during final manuscript assembly. Exact item-level denominators, score distributions, medians, interquartile ranges, and round-specific voting records therefore cannot be verified from the surviving files and have not been reconstructed from memory or generated post hoc. Supplementary Table S4 reports only the final disposition of retained items; the prespecified consensus rule is described above.

**Supplementary Table S4. Final disposition of retained consensus items**

| Final item | Short topic | Final status |
| --- | --- | --- |
| Expert Consensus Statement 1 | Four treatment-target dimensions | Retained |
| Expert Consensus Statement 2 | Four dimensions may overlap | Retained |
| Expert Consensus Statement 3 | Framework is not a predictive model | Retained |
| Expert Consensus Statement 4 | MAS cannot determine treatment target alone | Retained |
| Recommendation 1 | Goal-centered multidimensional assessment | Retained |
| Recommendation 2 | Noninvasive BCI as upper-limb adjunct | Retained |
| Recommendation 3 | BCI-FES as a closed-loop option | Retained |
| Expert Consensus Statement 5 | BCI metrics are not surgical thresholds | Retained |
| Recommendation 4 | DNB for reversible decision support | Retained |
| Expert Consensus Statement 6 | DNB as reversible clinical simulation | Retained |
| Expert Consensus Statement 7 | DNB does not predict all long-term outcomes | Retained |
| Recommendation 5 | Peripheral nerve surgery: patient selection | Retained |
| Expert Consensus Statement 8 | Goal of peripheral nerve surgery | Retained |
| Expert Consensus Statement 9 | Tone reduction is not a functional surrogate | Retained |
| Recommendation 6 | Structural reconstruction for fixed restriction | Retained |
| Expert Consensus Statement 10 | Tendon-transfer patient selection | Retained |
| Recommendation 7 | Individualized combined reconstruction | Retained |
| Expert Consensus Statement 11 | No fixed combined regimen | Retained |
| Expert Consensus Statement 12 | Procedure-specific postoperative motor retraining | Retained |
| Expert Consensus Statement 13 | Postoperative BCI is not standard care | Retained |
| Expert Consensus Statement 14 | Multidimensional outcome assessment | Retained |
| Expert Consensus Statement 15 | Neurophysiologic metrics as supplementary outcomes | Retained |

Items that failed to meet the prespecified criteria after the second vote were excluded before finalization. Because the raw item-level voting export and discarded-draft voting details were not recoverable, the exact number, wording, voting round, and numerical distribution of excluded draft items are not reconstructed in this supplement.

# Supplementary Table S5. Minimum documentation for diagnostic motor nerve block (DNB)

| Domain | Minimum documentation | Rationale |
| --- | --- | --- |
| Decision question | Prespecified clinical question and patient-centered goal that the block is intended to clarify. | Prevents interpretation of DNB as an unstructured positive/negative test. |
| Target | Target motor nerve/branch and intended muscle group(s). | Links the block to the hypothesized source of abnormal neural drive. |
| Technique | Guidance method, local anesthetic, procedural approach, and relevant safety considerations. | Allows interpretation and reproducibility without imposing a universal dose. |
| Timing | Time from injection to post-block assessment. | Immediate effects depend on the assessment window. |
| Matched examination | Same posture/task conditions before and after DNB; slow and fast passive ROM when relevant. | Improves within-patient comparability. |
| Active function | Direction, amplitude, and selectivity of active movement; target goal-related task. | Separates reduction in abnormal drive from meaningful functional change. |
| Residual limitation | Persistent fixed restriction, weakness, antagonist imbalance, or other limitation revealed after the block. | Supports treatment-target reassessment and combined/staged planning. |

# Supplementary Appendix S1. Exact archived PubMed search strings

**Comprehensive assessment and pathophysiologic components**

(("Stroke"[Mesh] OR "Brain Ischemia"[Mesh] OR "Intracranial Hemorrhages"[Mesh] OR stroke*[Title/Abstract] OR poststroke[Title/Abstract] OR "post-stroke"[Title/Abstract]) AND ("Muscle Spasticity"[Mesh] OR spastic*[Title/Abstract] OR "spastic paresis"[Title/Abstract] OR "muscle overactivity"[Title/Abstract] OR "abnormal muscle overactivity"[Title/Abstract]) AND ("Modified Ashworth Scale"[Title/Abstract] OR "Tardieu Scale"[Title/Abstract] OR "Goal Attainment Scaling"[Title/Abstract] OR contracture[Title/Abstract] OR "selective motor control"[Title/Abstract] OR "dynamic electromyography"[Title/Abstract] OR "gait analysis"[Title/Abstract])) AND ("2016/01/01"[Date - Publication] : "2026/08/30"[Date - Publication])

**Noninvasive BCI motor rehabilitation**

(("Stroke"[Mesh] OR "Brain Ischemia"[Mesh] OR "Intracranial Hemorrhages"[Mesh] OR stroke*[Title/Abstract] OR poststroke[Title/Abstract] OR "post-stroke"[Title/Abstract]) AND ("Brain-Computer Interfaces"[Mesh] OR "brain-computer interface*"[Title/Abstract] OR "brain computer interface*"[Title/Abstract] OR "brain-machine interface*"[Title/Abstract])) AND ("2016/01/01"[Date - Publication] : "2026/08/30"[Date - Publication])

**BCI-FES closed-loop training**

(("Stroke"[Mesh] OR "Brain Ischemia"[Mesh] OR "Intracranial Hemorrhages"[Mesh] OR stroke*[Title/Abstract] OR poststroke[Title/Abstract] OR "post-stroke"[Title/Abstract]) AND ("Brain-Computer Interfaces"[Mesh] OR "brain-computer interface*"[Title/Abstract] OR "brain computer interface*"[Title/Abstract] OR "brain-machine interface*"[Title/Abstract]) AND ("Electric Stimulation Therapy"[Mesh] OR "functional electrical stimulation"[Title/Abstract] OR "functional electric stimulation"[Title/Abstract] OR FES[Title/Abstract])) AND ("2016/01/01"[Date - Publication] : "2026/08/30"[Date - Publication])

**Diagnostic motor nerve block**

(("Stroke"[Mesh] OR "Brain Ischemia"[Mesh] OR "Intracranial Hemorrhages"[Mesh] OR stroke*[Title/Abstract] OR poststroke[Title/Abstract] OR "post-stroke"[Title/Abstract]) AND ("Muscle Spasticity"[Mesh] OR spastic*[Title/Abstract] OR "spastic paresis"[Title/Abstract] OR "muscle overactivity"[Title/Abstract] OR "abnormal muscle overactivity"[Title/Abstract]) AND ("diagnostic motor nerve block"[Title/Abstract] OR "diagnostic nerve block"[Title/Abstract] OR "motor nerve block"[Title/Abstract] OR "selective motor nerve block"[Title/Abstract])) AND ("2016/01/01"[Date - Publication] : "2026/08/30"[Date - Publication])

**Selective/hyperselective peripheral nerve surgery**

((("Stroke"[Mesh] OR "Brain Ischemia"[Mesh] OR "Intracranial Hemorrhages"[Mesh] OR stroke*[Title/Abstract] OR poststroke[Title/Abstract] OR "post-stroke"[Title/Abstract]) OR ("Muscle Spasticity"[Mesh] OR spastic*[Title/Abstract] OR "spastic paresis"[Title/Abstract] OR "muscle overactivity"[Title/Abstract] OR "abnormal muscle overactivity"[Title/Abstract])) AND ("selective neurotomy"[Title/Abstract] OR "selective neurectomy"[Title/Abstract] OR "hyperselective neurectomy"[Title/Abstract] OR "hyper-selective neurectomy"[Title/Abstract] OR "selective peripheral neurotomy"[Title/Abstract] OR "tibial neurotomy"[Title/Abstract])) AND ("2016/01/01"[Date - Publication] : "2026/08/30"[Date - Publication])

**Tendon and soft-tissue reconstruction**

(("Stroke"[Mesh] OR "Brain Ischemia"[Mesh] OR "Intracranial Hemorrhages"[Mesh] OR stroke*[Title/Abstract] OR poststroke[Title/Abstract] OR "post-stroke"[Title/Abstract]) AND (("Muscle Spasticity"[Mesh] OR spastic*[Title/Abstract] OR "spastic paresis"[Title/Abstract] OR "muscle overactivity"[Title/Abstract] OR "abnormal muscle overactivity"[Title/Abstract]) OR contracture[Title/Abstract]) AND ("Tendon Transfer"[Mesh] OR "tendon transfer*"[Title/Abstract] OR "tendon lengthen*"[Title/Abstract] OR "Achilles tendon lengthen*"[Title/Abstract] OR "soft tissue release"[Title/Abstract] OR "muscle release"[Title/Abstract] OR tenotomy[Title/Abstract] OR "myotendinous lengthen*"[Title/Abstract])) AND ("2016/01/01"[Date - Publication] : "2026/08/30"[Date - Publication])

**Post-reconstruction motor relearning and rehabilitation**

(("Stroke"[Mesh] OR "Brain Ischemia"[Mesh] OR "Intracranial Hemorrhages"[Mesh] OR stroke*[Title/Abstract] OR poststroke[Title/Abstract] OR "post-stroke"[Title/Abstract]) AND (("selective neurotomy"[Title/Abstract] OR "selective neurectomy"[Title/Abstract] OR "hyperselective neurectomy"[Title/Abstract] OR "hyper-selective neurectomy"[Title/Abstract] OR "selective peripheral neurotomy"[Title/Abstract] OR "tibial neurotomy"[Title/Abstract]) OR ("Tendon Transfer"[Mesh] OR "tendon transfer*"[Title/Abstract] OR "tendon lengthen*"[Title/Abstract] OR "Achilles tendon lengthen*"[Title/Abstract] OR "soft tissue release"[Title/Abstract] OR "muscle release"[Title/Abstract] OR tenotomy[Title/Abstract] OR "myotendinous lengthen*"[Title/Abstract])) AND (rehabilitation[Title/Abstract] OR "motor relearning"[Title/Abstract] OR "motor learning"[Title/Abstract] OR "task-specific training"[Title/Abstract] OR "task-oriented training"[Title/Abstract] OR "postoperative rehabilitation"[Title/Abstract])) AND ("2016/01/01"[Date - Publication] : "2026/08/30"[Date - Publication])

# Supplementary Appendix S2. Final-reference identification audit

The final bibliography contains 46 references. Exact normalized-title matching against the archived electronic datasets identified 20 references in the electronic evidence pool (19 in PubMed and 2 in CNKI, with one reference present in both). The other 26 references were not present in the archived electronic pool and are therefore classified as supplementary identification through citation tracking and/or expert nomination.

Direct electronic-pool reference numbers: 4, 7, 8, 9, 10, 12, 13, 14, 15, 17, 19, 21, 23, 29, 30, 32, 37, 39, 41, 44.

Supplementary-identification reference numbers: 1, 2, 3, 5, 6, 11, 16, 18, 20, 22, 24, 25, 26, 27, 28, 31, 33, 34, 35, 36, 38, 40, 42, 43, 45, 46.
